# Generation and screening of a comprehensive *Mycobacterium avium* subsp. *paratuberculosis* transposon mutant bank

**DOI:** 10.3389/fcimb.2014.00144

**Published:** 2014-10-15

**Authors:** Govardhan Rathnaiah, Elise A. Lamont, N. Beth Harris, Robert J. Fenton, Denise K. Zinniel, Xiaofei Liu, Josh Sotos, Zhengyu Feng, Ayala Livneh-Kol, Nahum Y. Shpigel, Charles J. Czuprynski, Srinand Sreevatsan, Raúl G. Barletta

**Affiliations:** ^1^School of Veterinary Medicine and Biomedical Sciences, University of NebraskaLincoln, NE, USA; ^2^Department of Veterinary Population Medicine, University of Minnesota, St. PaulMN, USA; ^3^School of Veterinary Medicine, University of WisconsinMadison, WI, USA; ^4^The Koret School of Veterinary Medicine, The Hebrew University of JerusalemRehovot, Israel

**Keywords:** Johne's Disease, *Mycobacterium paratuberculosis*, transposon, mutant bank, bovine macrophages

## Abstract

*Mycobacterium avium* subsp. *paratuberculosis* (MAP) is the etiologic agent of Johne's Disease in ruminants. This enteritis has significant economic impact and worldwide distribution. Vaccination is one of the most cost effective infectious disease control measures. Unfortunately, current vaccines reduce clinical disease and shedding, but are of limited efficacy and do not provide long-term protective immunity. Several strategies have been followed to mine the MAP genome for virulence determinants that could be applied to vaccine and diagnostic assay development. In this study, a comprehensive mutant bank of 13,536 MAP K-10 Tn*5367* mutants (*P* > 95%) was constructed and screened *in vitro* for phenotypes related to virulence. This strategy was designated to maximize identification of genes important to MAP pathogenesis without relying on studies of other mycobacterial species that may not translate into similar effects in MAP. This bank was screened for mutants with colony morphology alterations, susceptibility to D-cycloserine, impairment in siderophore production or secretion, reduced cell association, and decreased biofilm and clump formation. Mutants with interesting phenotypes were analyzed by PCR, Southern blotting and DNA sequencing to determine transposon insertion sites. These insertion sites mapped upstream from the MAP1152-MAP1156 cluster, internal to either the Mod operon gene MAP1566 or within the coding sequence of *lsr2*, and several intergenic regions. Growth curves in broth cultures, invasion assays and kinetics of survival and replication in primary bovine macrophages were also determined. The ability of vectors carrying Tn*5370* to generate stable MAP mutants was also investigated.

## Introduction

*Mycobacterium avium* subsp. *paratuberculosis* (MAP) is the etiologic agent of Johne's Disease (JD) in ruminants. This enteritis has significant economic impact and worldwide distribution (Sweeney, 1996). In the United States, annual losses to the dairy industry have been estimated from 250 million (Ott et al., 1999) to $1.5 billion (Stabel, 1998). Vaccination is one of the most cost effective disease control measures. Unfortunately, though there are JD vaccines that reduce clinical disease and shedding, their efficacies are limited and none afford long-term protective immunity. For example in the United States, Mycopar® (Boehringer Ingelheim Vetmedica, Inc.) is the only licensed vaccine against JD. However, this vaccine is derived from *M. avium* Strain 18 (Bastida and Juste, 2011), and therefore does not have an optimal antigenic repertoire. Another bacterin, Silirum® (Zoetis Animal Health) is being tested in Australia and approved for limited use. It is a heat-killed MAP vaccine strain with improved safety. This formulation may possess a better antigenic repertoire but heat-killing may reduce efficacy. Neoparasec® (Rhone-Merieux) contains the live-attenuated MAP strain 316F while Gudair® (Zoetis Animal Health) is heat-killed 316F and licensed for use in sheep and goats. However, current vaccines cannot distinguish vaccinated from infected animals, thus compromising JD diagnostic tests (Hines et al., 2014), and strain 316F was generated in the 1920's by random attenuation procedures (e.g., passages on ox bile) and their attenuating mutations are only now being investigated (Bull et al., 2013). In last analysis, a vaccine of high efficacy is needed for an effective control of JD (Lu et al., 2013).

The MAP wild type strain K-10 genome has been sequenced, annotated and reannotated by optical mapping (Li et al., 2005; Wu et al., 2009). The updated K-10 genome is represented by a circular map of 4,829,781 bp encoding 4350 open reading frames (ORFs) with 69.3% GC content. In this genome, about 60% of the ORFs have known homologs in databases but only 30% have definitive function predictions (Bannantine et al., 2012). Several strategies have been followed to mine the MAP genome for virulence determinants or antigens of diagnostic importance (Bannantine and Paustian, 2006; Cho et al., 2007; Li et al., 2007). One approach relied on identifying MAP genes with known *M. tuberculosis* (MTB) homologs or orthologs (Sampson et al., 2004; Bach et al., 2006). For example, based on the attenuation of MTB *leuD* mutants (Sampson et al., 2004), the corresponding MAP *leuD* mutants were constructed by allelic exchange, characterized by carbon and nitrogen source utilization, showed to be attenuated in mice (Chen et al., 2012a,b), and provide protection against wild type challenge in goats (Faisal et al., 2013). Indeed, differences in gene organization may lead to context-dependent function: e.g., even homologous genes in MAP and *M. avium* subsp. *hominissuis* (MAH) may play different roles in pathogenesis (Wu et al., 2006). In addition, genomic differences may be associated with specific hosts (Bannantine et al., 2012). Thus, strategies based on gene homology are not comprehensive. We have previously demonstrated that transposon Tn*5367* inserts relatively random into the MAP genome (Harris et al., 1999). Another study generated a mutant bank of 5060 K-10 mutants (*P* > 70%) (Shin et al., 2006). Based on bioinformatic analysis, 11 mutants were selected for mouse infection experiments (Shin et al., 2006), identifying potential virulence genes (*gcpE*. *pstA*. *kdpC*. *papA2*. *impA*. *umaA1*, and *fabG2_2*). In this study, we constructed an expanded and comprehensive bank of 13,536 K-10 mutants (*P* > 95%) and performed phenotypic screens *in vitro*. We believe this strategy maximizes possible hits in genes important to MAP pathogenesis without relying on studies on other mycobacterial species that may not always translate into similar effects in MAP. We also tested the utility of vectors carrying Tn*5370* (McAdam et al., 2002) to generate stable MAP mutants.

## Materials and methods

### Bacterial strains, growth conditions and recombinant DNA

A virulent clinical isolate of MAP (original stock of strain K-10) was used throughout the study (Foley-Thomas et al., 1995). MAP cultures were grown standing with occasional shaking at 37°C in complete Middlebrook 7H9 media (MOADC-Plus) as previously described (Harris et al., 1999); see also Section Transposon mutagenesis below. Depending on the experiment, the supplementation with mycobactin J (Allied Monitor, Fayette, MO) varied from 0.5 to 2.0 μg/ml. For further characterization of the selected mutants, we used this media without L-tryptophan and casamino acids containing vitamins (MOADC). *E. coli* DH5α cells, used as cloning hosts, were grown on Luria-Bertani agar or broth supplemented with 50 μg/ml kanamycin (Kan). The conditionally replicating (temperature sensitive) recombinant mycobacteriophage phAE94 (Bardarov et al., 1997) was used to deliver the transposon Tn*5367*, and signature-tag TM4 phage derivatives of the thermosensitive vector phAE87 were used to deliver Tn*5370* (Bardarov et al., 1997; Cox et al., 1999; McAdam et al., 2002). Phage vectors were propagated in *M. smegmatis* (Msmeg) mc^2^155 at 30°C as described previously (Bardarov et al., 1997). The Kan-resistance (Kan^r^) Tn*5367* and the hygromycin (Hyg)-resistant (Hyg^r^) Tn*5370* transposons are derived from the insertion sequence IS*1096* from Msmeg (Cirillo et al., 1991).

### Transposon mutagenesis

MAP cultures were grown to ca. 1.5 × 10^8^ colony forming units (CFU)/ml (OD_600_ 0.38 to 0.75). Cultures (50 ml) were concentrated by centrifugation and resuspended in 1 ml of MP buffer (50 mM Tris-HCl, pH 7.6; 150 mM NaCl; 2 mM CaCl_2_). Bacteria and phage were incubated at the non-permissive temperature (37°C) and stop buffer was added as described previously (Bardarov et al., 1997; Harris et al., 1999). The addition of the stop buffer with sodium citrate helps to prevent further phage infections that may lead to multiple transposon insertions in a single strain. Under these conditions, the potential number of double insertions was shown to be at most 1 in 12 mutants (8.3%). Kan^r^ or Hyg^r^ colonies were selected on MOADC-Plus medium without Tween plus 15 g/l agar containing 50 μg/ml Kan or 75 μg/ml Hyg, supplemented with 0.4% Bacto Casamino Acids (Difco, Becton Dickinson, Franklin Lakes, NJ), and 40 μg/ml L-tryptophan. Transductants were isolated from these plates after 6–8 week incubation period at 37°C.

### Mutant screening and characterization assays

#### Visual screen for colony morphology mutants

To identify colony morphology, MAP mutant strains were grown individually in 96-well microtiter plates in MOADC-Plus fully-supplemented media with 0.5 μg/ml mycobactin J, as described above. Plates were photographed and pictures visually inspected for colony morphology alterations in the bottom of the corresponding wells. Colony morphology alterations were confirmed by further observations on the corresponding agar plates.

#### Susceptibility to D-cycloserine

Screen was accomplished by first determining the highest concentration of D-cycloserine (DCS) that would allow wild type growth to occur on MOADC-Plus fully-supplemented solid media (without Tween) for both MAP wild type K-10 and most randomly selected transposon mutants. All mutant strains were then individually replicated onto solid media without and with DCS at the established concentration. After 8 week incubation at 37°C, DCS susceptibility phenotypes were evaluated and classified as hypersusceptible (failed to grow or grew poorly on DCS), wild type (grew normally, similarly to K-10 on DCS), and resistant (grew faster than K-10 on DCS, reaching a larger colony size).

#### Miscellaneous screening procedures

To screen for mutants deficient in siderophore production or secretion, individual mutant colonies were grown on MOADC-Plus media, as previously described (Harris et al., 1999). A portion of the bacterial colony was then aseptically transferred onto modified Chrome azurol S (CAS) plates. This media consisted of 72.9 mg/l of hexadecyltrimethylammonium bromide (Sigma, St Louis, MO), 60.5 mg/l of CAS (Sigma), 10 ml/l of iron solution (1 mM FeCl_3_·6H_2_O, 10 mM HCl), 4.7 g/l of Middlebrook 7H9 broth base (Difco, Becton Dickinson), 2 ml/l glycerol and 15 g/l agar adjusted to pH 5.9. The transferred cell patches were incubated at 37°C for 2 weeks and visually screened for the absence of a yellowish halo around the colonies, as previously described (Schwyn and Neilands, 1987). The presence of a halo indicates that siderophores produced and secreted by the colony bind ferric iron in the media.

Biofilm production, thought to be linked to bacterial virulence was also examined. Bacteria were grown in MOADC-Plus broth using plastic tubes and for several weeks. The presence of biofilm was established by visual observation at the sides of the tubes and at the liquid-air interphase. Additional miscellaneous screens are described in the Supplementary Material.

#### Mutant characterization in BoMac cells

BoMac (Stabel and Stabel, 1995) monolayers adherent to glass coverslips were infected with approximately the same inoculum (MOI 10:1) in RPMI standard tissue culture medium supplemented with 2% fetal bovine serum for 2–3 h (invasion incubation) at 39°C. Cells were washed, coverslips removed, fixed and acid-fast stained. For each experimental group, a minimum of at least 100 cells were examined at 1000X magnification under a microscope to determine the percentage of macrophages containing acid-fast bacilli and to enumerate the number of bacteria per macrophage. Bacterial growth at various times post-infection (e.g., Day 0 after invasion incubation and Day 4) was determined by colony counts and BACTEC radiometric assays. Monolayers were lysed with 0.05% sodium dodecyl sulfate for 30 min, and lysates were inoculated into BACTEC 12B vials containing ^14^C-palmitate supplemented with 2.0 μg/ml of mycobactin J and 1 ml of egg yolk. The BACTEC 460 instrument (Becton Dickinson Diagnostic Instrument Systems, Sparks, MD) was utilized to determine the release of ^14^CO_2_ from palmitate that was recorded by the instrument as a growth index (recorded every 24 h until the cumulative index reached 2000 in about 20 days). These indices were converted into viable cell numbers per well (CFUs) using a previously described formula (Lambrecht et al., 1988). The full validation of this method is given elsewhere (Zhao et al., 1997, 1999). As needed, results were confirmed by CFU determinations on MOADC-Plus agar with 2.0 μg/ml mycobactin J.

### Oligonucleotide primers and PCR

The oligonucleotide primers used in this study are listed in Table 1. The general conditions for the PCR reactions were: 0.03 ml reaction mixture volume containing 170 nM of each specific primer pair, in the presence of 1× NH4-based Reaction Buffer (Bioline USA, Inc., Taunton, MA), 1.7 mM MgCl_2_, 8.3% (v/v) DMSO, 0.25 mM deoxynucleoside triphosphates and 1.5 units of Biolase™ DNA Polymerase (Bioline USA, Inc.). Thermo cycler settings of 95°C for 5 min, followed by 30 cycles of 95°C for 30 s, 61°C for 45 s, 72°C for 1 min and a final extension at 72°C for 7 min.

**Table 1:** **List of primers used in this study**.

**Primer**	**Sequence (5′–3′)**
2E11-IP-F	GCTGCAGCAACCAGCCGA
2E11-IP-R	CCACCGTCACCGCAGGTAGA
3H4-F	TCGCGGTCCTCGTATTCGCT
3H4-R	TGTCGGACGTGTCCGGTCAC
4H2-F	TCAAGTGGGTTGTGCCCCGT
4H2-R	GCTACCCAGGAGACGCGCCT
22F4-F	GTATCGACCGGTTGTTGATG
22F4-R	GCCGATGTAGTTGTGGTTGA
40A9-IP1-F	GGATGAATCCTCGGCCTTGG
40A9-IP1-R	CCGACGGCGTAGTCTGCAAT
AMT32	CTCTTGCTCTTCCGCTTCTTCTCC
AMT152	TTGCTCTTCCGCTTCTTCT
Hygro-F	ATAGACGTCGGTGAAGTCGACGAT
Hygro-R	GAATCCCTGTTACTTCTCGACCGT
IS900-F	GGATGGCCGAAGGAGATTGG
IS900-R	GCAGCTCGACTGCGATGTCA
IS1096-F	TCGCACTTGACGGTGTA
IS1096-R	GTCGGCTCATCGAACAT
Kan-F	TCGAGGCCGCGATTAAATTCCACC
Kan-R	ATTCATTCGTGATTGCGCCTGAGC
MAP1566-F	GCTCTAGAGCTGGCATCAGGGCACTCAAGAAA
MAP1566-R	CCCAAGCTTGGGTATTCGCTGCACAGCATGTCAGGT
RS6-4	GTAATACGACTCACTATAGGGCNNNNCATG
SP1	TGCAGCAACGCCAGGTCCACACT
SP2	CTCTTGCTCTTCCGCTTCTTCTCC
STM5370-1	TGCTAGGCGTCGGCCATTAGC
T7	TAATACGACTCACTATAGGG

### Southern blotting

Chromosomal DNA was isolated from wild type and mutant stains. To determine the presence of a transposon insertion, for mutants derived from Tn*5367*, DNA samples were digested with *EcoRI*, which does not cut within this transposon. For Tn*5370* derivatives, DNA samples were digested with *NheI* and *NdeI*, which do not cut within this transposon. Digested DNA fragments from both wild type and mutant strains were electrophoresed on a standard agarose gel and transferred to a nylon membrane (Osmonics, Inc., GE Water and Process Technologies, Trevose, PA). Probes were prepared by PCR-amplification using specific primers (Table 1): IS*1096* probe was amplified from Msmeg genomic DNA with IS1096-F and IS1096-R and IS*900* probe was amplified from MAP genomic DNA using IS900-F and IS900-R. The Kan-resistant *aph* gene probe for Tn*5367* was amplified from pMV262 (Connell et al., 1993), or plasmids derived there from, with Kan-F and Kan-R. The Hyg-resistant *hyg* gene probe for Tn*5370* was amplified from pYUB854 (Bardarov et al., 2002), or plasmids derived there from, using Hygro-F and Hygro-R. Probes were labeled with α-[32P]-dCTP (3000 Ci/mmol) using the *Redi*prime DNA Labeling System (Amersham-Pharmacia Biotech, Inc., Molecular Dynamics Div., Piscataway, NJ) as described by the manufacturer. Hybridization and detection were done as described previously with minor modifications (Feng et al., 2002). Southern blot images were digitally captured and processed only to enhance brightness and contrast (Bio-Rad Imaging system, Hercules, CA).

### Sequencing of the transposon-insertion site

In order to locate the transposon insertion sites in the K-10 genome, DNA was amplified for sequencing analysis by a nested PCR as previously described (McAdam et al., 2002). A new primer designated STM5370-1, common to both transposons, was used in place of the original SP1 primer due to differences in the Tn*5367* and Tn*5370* transposon sequences. This primer was designed based on sequence information of Tn*5370* provided Dr. Jeffrey Cirillo (Texas A&M Health Science Center) that was further supplemented by our own sequencing studies. The first PCR used the following primers: RS6-4 (degenerate) and STM5370-1. The variable size PCR products (1 μl) from the first cycle were used in a second PCR cycle with primers T7 and SP2 that are internal to the first cycle PCR product. These final PCR products were purified by Wizard PCR Preps DNA Purification System (Promega, Madison, WI). The samples were run on 1.5% agarose gel electrophoresis to recover the most concentrated DNA bands and purified by the GeneClean® III Kit (Qbiogene, MP Biomedicals, LLC, Solon, OH). DNA samples were sent to The University of Nebraska Genomics Core Research Facility for sequencing, using AMT152 as primer (Shin et al., 2006). Sample runs were performed on a Beckman-Coulter CEQ8000 or CEQ2000XL 8-capillary DNA sequencer using dye-terminator chemistry to provide 500–650 bp of sequence.

### Growth curves in broth cultures

MAP strains were grown to late exponential phase (OD_600_ between 1.0 and 1.5) at 37°C from glycerol stocks in MOADC media (1.0 μg/ml mycobactin J), appropriately supplemented with antibiotics as needed. Applicable culture volumes were inoculated into 45 ml of MOADC media in T75 flasks with vented caps so that the initial OD_600_ was approximately 0.05. Each culture was incubated at 37°C standing with OD_600_ readings being taken on days 0, 3, 5, 7, 10, 12, 14, 17, 21, 24, and 28 around the same time each day. CFU counts were also determined on MOADC agar.

### Primary bovine macrophage assay

Monocyte-derived macrophages (MDMs) from a JD negative dairy cow were elutriated and enriched as described (Lamont and Sreevatsan, 2010). Incubations were conducted in a 37°C humidified chamber containing 5% CO_2_. Approximately 2.0 × 10^7^ MDMs were seeded into separate T25 flasks and allowed to adhere for 2 h. MDMs were washed thrice to remove non-adherent cells and fresh RPMI 1640 supplemented with 2% autologous serum was added prior to MAP infection. Subcultured MAP (K-10 wild type or mutant strains) were grown to an OD_600_ of 0.5 (approximately 1.0 × 10^8^ cells/ml) and pelleted at 8000 × g for 10 min. The pellet was washed thrice using 1X phosphate buffered saline (PBS), resuspended in RPMI 1640 containing 2% autologous serum (MOI 10:1), and vortexed for 5 min. MAP cells were repeatedly drawn through a 21-gage needle to break bacterial clumps. Cells were incubated at 37°C for 5 min to sediment any remaining clumps and only the upper 2/3rd volume was used for infection. MDMs were infected with MAP for 2 h (invasion incubation), washed thrice with 1X PBS to remove non-adherent bacteria, and recovered in RPMI 1640 supplemented with 2% autologous serum for the following post-infection time points: 0, 2, 6, 12, 24, and 48 h after the completion of the invasion incubation period. Upon completion of each post-infection time point, MDMs were washed thrice in 1X PBS and incubated with 0.01% Triton X-100 in PBS for 5 min at room temperature. The lysate was subjected to differential centrifugation at 388 ×g and 8000 × g for 5 min each to remove MDMs and pellet bacteria, respectively. MAP pellets were washed thrice in 1X PBS at 8000 ×g for 5 min to remove any remaining detergent, and resuspended in 1.0 ml of 1X PBS. All time points were assayed in triplicate. MAP inoculum and post-infection time points were separately stained using the Baclight Live/Dead kit (Invitrogen, Carlsbad, CA) and gated for live cells on a flow cytometer per manufacturer's instructions as described (Lamont and Sreevatsan, 2010).

### Calculation of growth parameters and statistical analysis

Statistical analyses to determine *P*-values presented in the text and figures were performed using the Statistical Analysis System (SAS) software version 9.1.3, 2004 (SAS Institute) using the Mixed Procedure Subroutine. *Post-hoc* comparisons were carried out using Tukey adjusted *P*-values. For calculations involving ODs and CFUs, logarithmic transformations were applied.

### Nucleotide sequence accession numbers

The DNA sequences of transposons Tn*5367* (Cirillo, J. D., McAdam, R. A., Weisbrod, T. R., Barletta, R. G. and Jacobs, W. R. Jr.) and Tn*5370* (Cirillo, J. D., Rathnaiah, G., McAdam, R. A., Weisbrod, T. R., Barletta, R. G. and Jacobs, W. R. Jr.) have been assigned GenBank accession numbers KM232614 and KM232615, respectively.

## Results

### Generation and screening of a transposon mutant bank

To investigate virulence factors of MAP, a transposon mutant bank of MAP wild type K-10 was constructed. The transposon Tn*5367* carrying a Kan resistance marker (Bardarov et al., 1997) randomly inserted into the bacterial chromosome by transposition from a thermosensitive phage delivery vector. Nine independent infections (8 with phage and 1 control with no phage) were carried out to ensure that sibling mutants were minimally represented. A total of 13,536 Kan-resistant mutants were collected and stored individually in an indexed collection of 141 96-well plates stored at −80°C. Each mutant is identified by the plate number and the coordinates of the well (a letter for the row and a number for the column): e.g., mutant 4H2 is stocked in plate 4 and the well that intersects row H and column 2 in a 96-well microtiter plate. In addition, to facilitate screening procedures, pools of 96 or 24 mutants were also stocked so they could be easily referred to individual mutants in the indexed collection. Assuming random transposition and 4500 target genes with single transposon insertions [ln (1 − *P*) = *N* × ln(1-1/4500)], this number of mutants yields a *P*-value of approximately 95% for insertions in non-essential genes. The full library or a subset of strains were subjected to various individual screens to find strains of interest for studying MAP physiology and pathogenesis, and principally to search for attenuated strains that could be used as first-generation vaccine candidates in animal trials. A set of 123 strains, fairly well distributed through the various plates, were analyzed to show that the library was composed of strains with diverse phenotypes (Table S1).

#### Visual screen for colony morphology mutants

**Figure 1:**
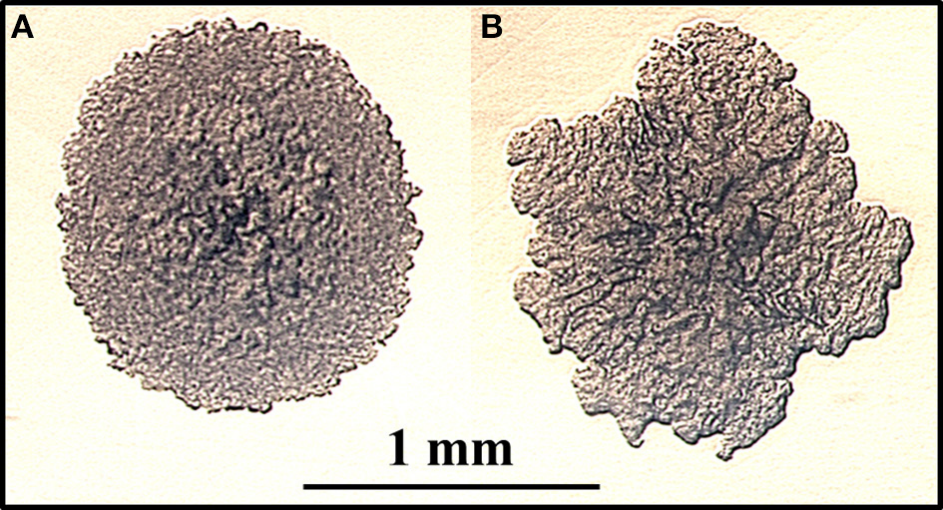
**(A,B) Identification of a MAP mutant with altered colony morphology**. MAP strains were grown on fully supplemented MOADC-Plus medium until colonies were approximately 1 mm in diameter (see scale bar) and photographed at 100X magnification. Colony morphologies for the wild type strain K-10 **(A)** and the Tn*5367* transposon mutant 4H2 **(B)** are displayed.

Colony morphology of microorganisms is well known to influence virulence, drug susceptibility and macrophage survival (Reddy et al., 1996). To identify colony morphology of MAP mutants, each of the 13,536 mutant strains was grown on MOADC-Plus media, visually observed and photographed with a camera. Using this procedure, eight mutants were found that exhibited colony morphologies different from the wild type strain (1F3, 4H2, 16B11, 22F4, 65E9, 69D12, 73D7, and 84D12). In particular, 4H2 displayed highly irregular colony borders and a rougher surface appearance as compared to K-10 (Figure 1 and Figure S1A). In contrast, mutant 22F4 displayed a smooth colony morphotype (Figure S1C). These two mutant strains were further tested in BoMac cells (see Section Miscellaneous screening). It is noted that visual screens may depend on the media used and undoubtedly have a degree of subjectivity. Thus, less obvious but yet relevant differences in colony morphotype may not have been identified.

#### Susceptibility to DCS

DCS inhibits peptidoglycan biosynthesis in mycobacteria by the combined inhibition of D-alanine racemase (Alr) and D-alanine ligase (Chacon et al., 2002, 2009; Feng and Barletta, 2003; Halouska et al., 2014). Inactivation of the Msmeg *alr* gene results in a 500-fold reduction in the ability of Msmeg to survive within phagocytic cells and also determines a DCS hypersusceptible phenotype (Chacon et al., 2009). In addition, there are several potential alterations that can lead to DCS susceptibility or resistance including alterations of surface proteins that may also play a role in pathogenesis. To test this hypothesis in MAP, the entire mutant bank was screened for DCS susceptibility. First, it was determined that 20 μg/ml DCS was the maximum concentration that had no effect on the growth of K-10 and a subset of randomly picked transposon mutant strains. Using this approach, 98 mutant strains were found hypersusceptible to DCS. Sixty-three strains did not grow: e.g., colony morphotypes 1F3, 16B1, and 22F4 (Figures S1C,D); and normal colony morphotypes 12G8, 37D1, and 30H9. Thirty-five mutants grew poorly: e.g., normal colony morphotypes 3D4 and 23B5, but displayed normal growth without the drug. However, 15 DCS resistant mutants grew faster, as evidenced by expanded colony sizes: e.g., colony morphotypes 4H2 (Figures S1A,B), 65E9 and 69D12; and normal colony morphotype 40A9. The colony morphotypes 4H2 and 22F4 (see Section Visual screen for colony morphology mutants), as well as strains 30H9 and 40A9, were selected for further testing in BoMac cells (see Section Miscellaneous screening). In contrast, colony morphology mutants 73D7 and 84D12 displayed wild type susceptibility. In summary, this screen yielded 113 potentially attenuated strains, the greatest number among the assays performed.

#### Miscellaneous screening

**Figure 2:**
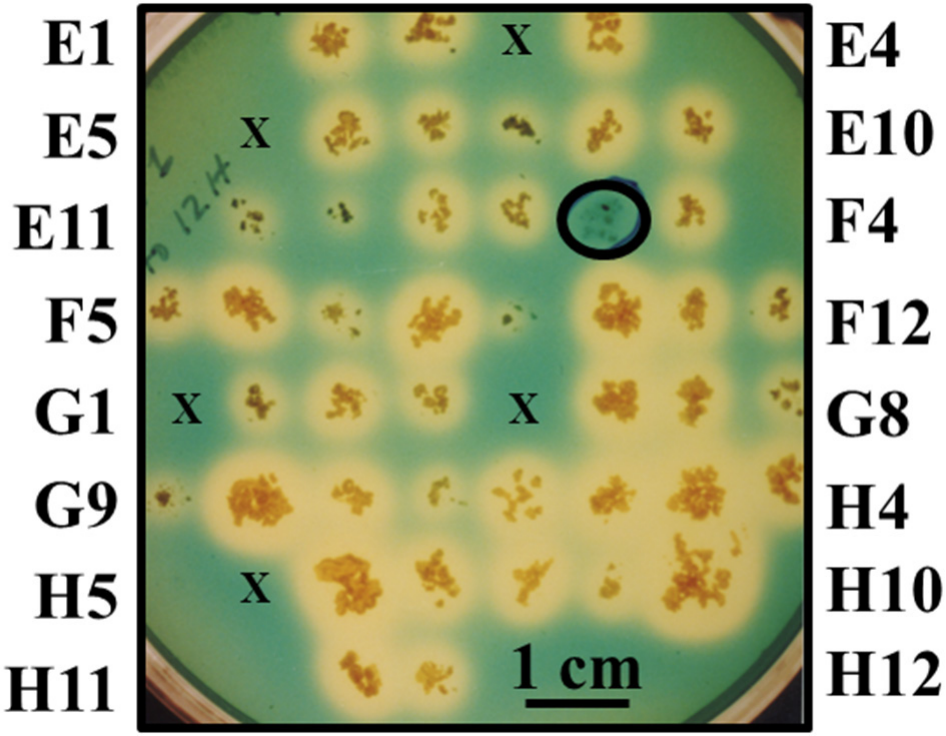
**Direct visual screen for MAP mutants unable to produce or secrete siderophores**. Mutants were grown on MOADC-Plus media for about 8 weeks, transferring a portion of the colony to Chrome azurol S plates since this media does not allow for growth. Labels for the outermost colonies are shown for each row and column (according to 96-well plate nomenclature) to designate individual mutants. Mutants that failed to grow initially are represented by an “×” and mutants that produced a halo are controls. A scale bar is provided to estimate halo size (blue to yellow color change when a strong chelating agent removes iron from the dye) after 2 weeks of incubation at 37°C. The circled colony without a halo is mutant 1F3 impaired in siderophore production or secretion.

Chromogenic screening of the entire mutant bank was performed to identify mutants unable to synthesize and/or secrete siderophores other than mycobactin. Since MAP is a known auxotroph for mycobactin, the goal of this experiment was to test the hypothesis that mycobacterial mutants unable to synthesize and/or secrete any other type of siderophore may be attenuated (Fiss et al., 1994). This method identified mutant 1F3 that grew significantly slower than K-10 in standard mycobactin J supplemented media did not produce a halo on the modified CAS medium plates (Figure 2). This phenotype suggests an impairment of the synthesis or transport of an alternative siderophore different from mycobactin. This mutant also displayed colony morphology alterations (see Section Visual screen for colony morphology mutants) and DCS hypersusceptibility (see Section Susceptibility to DCS). As stated in Section Generation and screening of a transposon mutant bank, the entire mutant bank, as well as 1F3, was stocked as a 96 well-plate culture. Replicate plating on MOADC-Plus for the CAS assay was conducted 6 months thereafter with all mutants, except 75, being viable. However, in a subsequent replication years later, mutant 1F3 grew poorly but in sufficient amounts to conduct a preliminary goat experiment when sent to collaborators in Israel (Livneh et al., 2005). Unfortunately, subcultures at both laboratories no longer grew.

Biofilm screening was also performed with a limited set of ca. 300 mutants to identify strains deficient in biofilm production (Figure S2). We identified mutants 2E11, 4E1, and 4E7 that were impaired in biofilm production. However, mutant 2E11 biofilm deficiency was due to poor growth in the assay. In contrast, mutant 4F3 produced more biofilm than the wild type strain. Additional screens identified mutants 3D4 and 3B11 with impaired cell association in BoMac cells (Figure S3), and strain 3D9 and 4E7 displayed impaired clump formation (Figure S4). All of these seven mutants did not display altered colony morphotypes. In addition, all of these mutants, except for 3D4, had normal DCS susceptibilities. Nonetheless, serendipitously, mutant 2E11 was selected for further studies as preliminary assays with MDMs indicated significant attenuation (see Section Interaction of wild type and mutant strains with primary bovine macrophages).

#### MAP mutant characterization in BoMac cells

**Figure 3:**
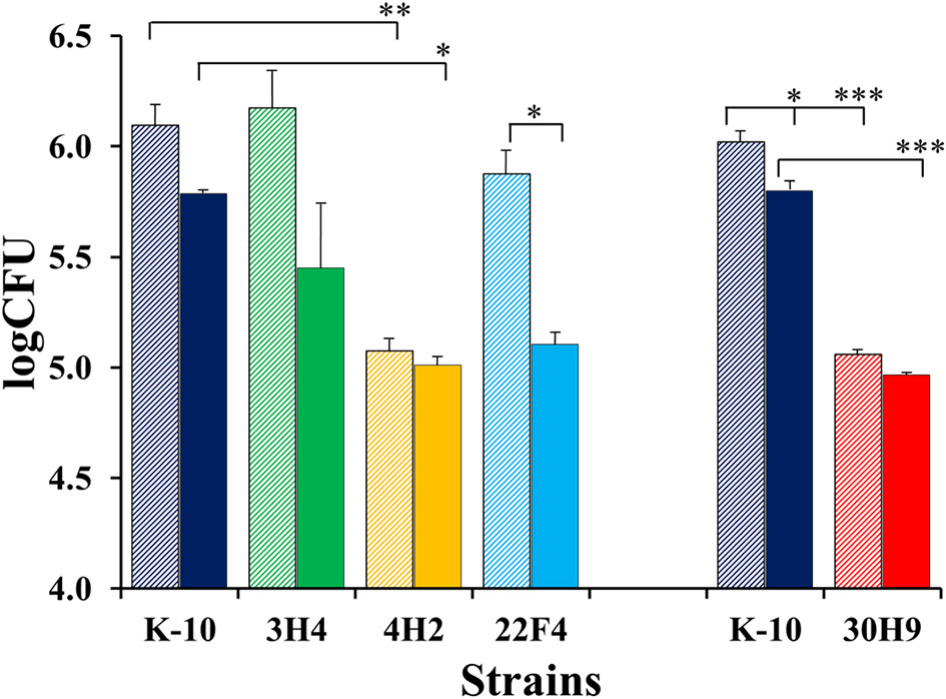
**Interaction of MAP wild type K-10 and mutant strains with the BoMac cell line**. Macrophage monolayers were infected with bacteria at a multiplicity of 10:1, incubated and lysed. Counts were taken at Day 0 (after invasion; stripe bars) and Day 4 (post-infection; solid bars). Bacilli were enumerated using the BACTEC radiometric assay system by recording growth indices every 24 h until this cumulative index reached 2000 (usually 20 days). These indices were converted into viable cell numbers per well (CFUs) using a previously described formula that has been extensively validated for MAP (Lambrecht et al., 1988; Zhao et al., 1997, 1999). Results represent means (*n* = 2) ± standard error of the mean. Statistical significance for pairwise determinations displayed by a line with tick marks is denoted with asterisks: ^*^P-value < 0.05, ^**^P-value < 0.01 and ^***^P-value < 0.001.

The invasion, survival and/or replication of selected MAP strains were first tested in the BoMac cell line, as these cells provide reproducibility over primary macrophages for the extended period of time that would be required to test a large number of mutants. Preliminary assessment of bacillary counts for representative strains was performed by microscopic examination of acid-fast stained monolayers on glass coverslips. Results were confirmed by CFU determination and BACTEC assays that were better suited for screening assays. Finally, the more promising mutants were screened in this fashion by BACTEC assays. Representative results are shown for two sets of independent experiments (Figure 3).

In the first set (Figure 3, left), K-10 was compared with 3H4 (with normal colony morphology, but unable to grow in DCS), 4H2 and 22F4. Results at Day 0 (after invasion incubation) showed that the invasiveness of 3H4 and 22F4 were similar to K-10 (*P* > 0.1), but 4H2 was significantly less invasive than the wild type strain K-10 (*P* < 0.01), and also less invasive than 3H4 (*P* < 0.01) and 22F4 (*P* < 0.05). Survivability for each strain was estimated by comparing the CFUs at Day 0 and Day 4: interaction of K-10 with BoMac cells resulted in decreased survival but the effect was not statistically significant (*P* > 0.1), reduced survivability was more pronounced and approached significance for 3H4 (*P* = 0.055), there was almost no change for 4H2 (*P* > 0.1), but reduction in CFUs was significant for 22F4 (*P* < 0.05). Comparison of the overall macrophage CFUs at Day 4 among all strains indicated that the 3H4 burden was not significantly different from K-10 (*P* > 0.1), while 22F4 showed a reduced burden but was not significantly different (*P* = 0.075), while 4H2 showed significantly less CFUs (*P* = 0.039).

Results for the second set (Figure 3, right), indicated that 30H9 was less invasive than K-10 (*P* < 0.001), but showed no significant difference (*P* > 0.1) in survivability from Day 0 to Day 4. In addition, 30H9 had a significantly lesser CFU burden than K-10 at Day 4 (*P* < 0.001). Notably, for this set, K-10 displayed a similar reduction in survivability from Day 0 to Day 4, but in this case, on account of more precise measurements and reduced number of means comparisons, the decrease was statistically significant (*P* < 0.05). In a separate assay, mutants 12G8 and 37D1 were also shown to be attenuated in BoMac cells. Mutant 23B5 displayed an attenuated phenotype when directly tested in MDMs infection assays (Table S1).

In summary, 4H2 and 30H9 were less invasive than the wild type K-10 strain, but had similar intracellular survival as K-10. In contrast, 22F4 was the only strain that was significantly less able to survive, by the end point, at Day 4 post-infection. The combined effects of invasion and survival, as indicated by the absolute CFU burdens as determined by the BACTEC assay, at Day 4, indicated that 4H2 and 30H9 were definitely attenuated in BoMac cells, predominantly by their reduced invasiveness. Thus, these two strains, once internalized at lower CFU burdens, persisted post-infection at approximately constant numbers. In contrast, 22F4 was attenuated because of its reduced survival, but the effect was of lesser degree. The normal colony morphotype mutant 3H4 behaved similarly to the wild type strain K-10.

### Further characterization of transposon mutants of interest

To further characterize mutants of interest, Southern hybridization analyses, and identification of transposon insertion sites by nested PCR sequencing were performed. Tests to identify specific transposon mutants in mixed infections were developed. In addition, these mutants were characterized by determination of growth curves in broth cultures and their interactions with primary bovine macrophages.

#### Southern hybridization

**Figure 4:**
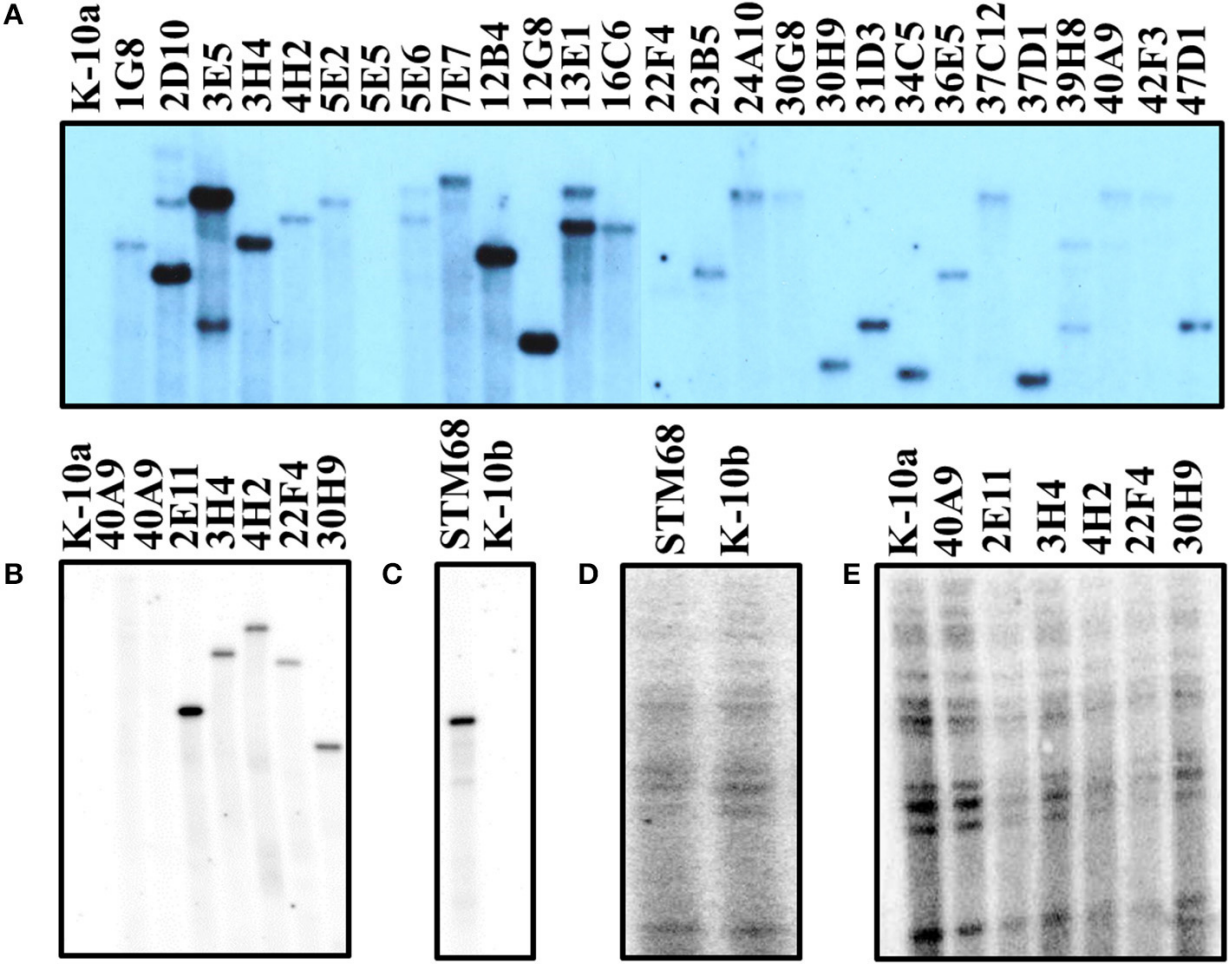
**(A–E) Southern blot analysis of MAP wild type K-10 and transposon insertion mutants**. DNA samples from Tn*5367* mutants and the wild type strain (K-10a) were digested with *Eco*RI **(A,B,E)**. DNA samples from the Tn*5370* mutant STM68 and the wild type strain (K-10b) were digested with *Nhe*I and *Nde*I **(C,D)**. DNA samples were run on agarose gels, transferred to a nylon membrane and hybridized with the IS*1096* probe to verify the presence of the transposon **(A–C)** and the IS*900* probe to confirm that the mutants were derived from MAP **(C,D)**. **(B–E)** are hybridization images generated from the same Southern transfer, while **(A)** was from an independent transfer.

To characterize the molecular events that gave rise to the mutant strains, Southern hybridizations were performed with various probes. Hybridization with a probe specific for the common transposon insertion sequence IS*1096* showed unique size bands for 33 mutant strains (not all positive strains shown in Figure 4; see Table S1), indicating the presence of a single copy of the corresponding transposon (Figures 4A–B). These results were confirmed by hybridization with a probe specific for either the Kan-resistant *aph* marker for Tn*5367* derivatives, or the Hyg-resistant *hyg* marker for Tn*5370* derivatives. Both probes yielded bands in the same gel positions as the IS*1096* probe (unpublished results). To verify that mutant strains were derived from K-10 and that no major molecular rearrangements have occurred, a probe specific for IS*900* was used. As expected, this analysis showed that all mutant strains analyzed had the same hybridization banding pattern as the wild type strain K-10 (Figures 4C–D). As mutants were directly analyzed from the original stocks (Figure 4A), we noted that 22F4 did not show a clear hybridization band with IS*1096*, and mutant 40A9 showed a very faint band. Upon replication to make large stocks for further analyses, mutant 22F4 clearly displayed the corresponding hybridization band, but 40A9 displayed no band, indicating the potential loss of the transposon insertion (Figure 4B). In addition, the original stock of mutant 5E5 did not display a hybridization band.

#### Transposon insertion site identification by sequencing of nested PCR amplicons

**Figure 5:**
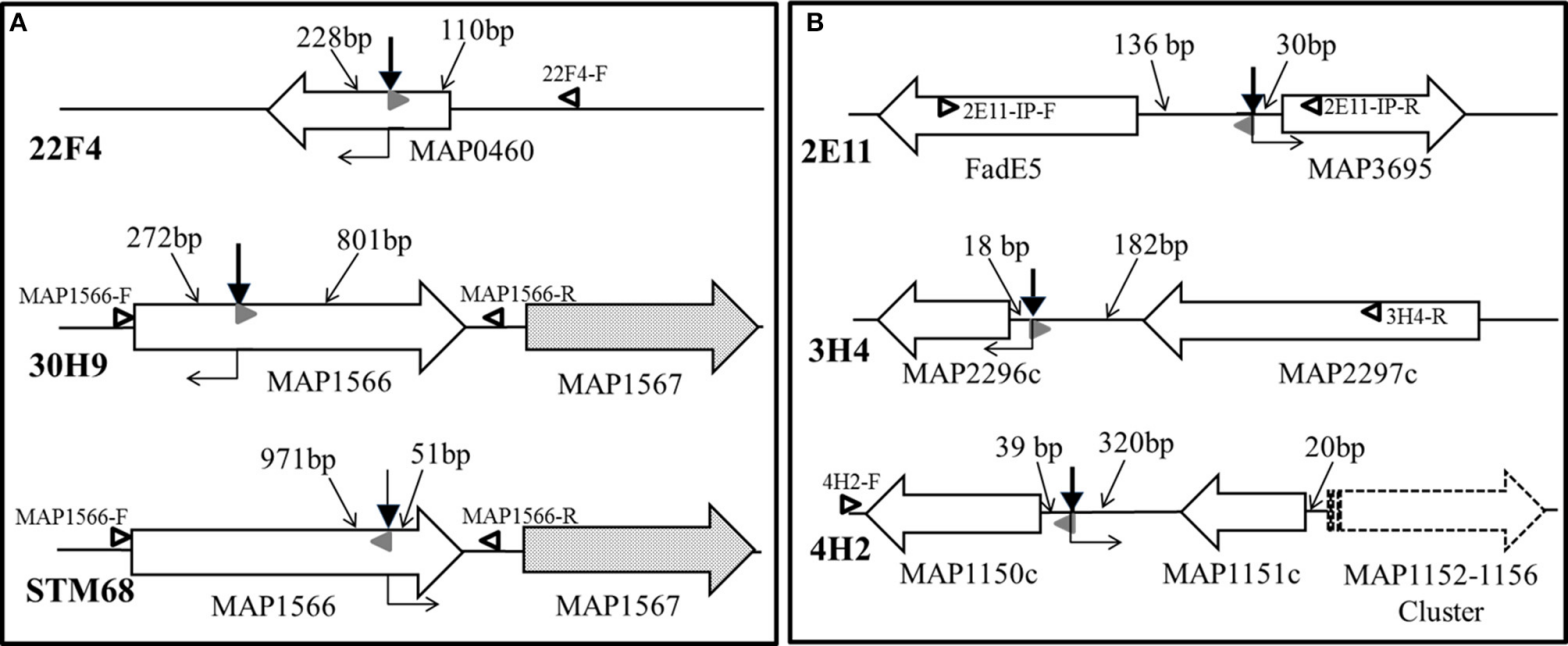
**(A,B) Transposon insertion sites of MAP mutants. Solid line wide arrows represent ORFs and indicate the direction of transcription**. Dotted fill wide arrows exemplify ORFs subjected to a potential polar effect due to the transposon insertion. The dashed line wide arrow represents a gene cluster transcribed in the same direction. Bold thick vertical arrows stand for Tn*5367* while the bold thin arrow symbolizes Tn*5370* insertion points. Regular thin arrows point to the DNA segments indicating distances (bp) between the transposon insertion point and the start or end of the corresponding ORF. Open black and solid gray (AMT32) sideway triangles represent primers used in PCR mutant identifications for mixed infection experiments (see Figure 6). Right angle bent arrows indicate the direction of transcription of the drug selection markers (*aph* in Tn*5367* derivatives; *hyg* in Tn*5370* derivative STM68) in the transposon. Diagram representing relevant genomic regions for mutants are represented: **(A)** 22F4, 30H9, and STM68; and **(B)** 2E11, 3H4, and 4H2.

The genomic regions containing transposon insertions of interest were amplified by nested PCR and sequenced as described (see Section Sequencing of the transposon-insertion site). Transposon insertion sites were identified by performing BLASTN identity searches of the MAP DNA sequences adjacent to transposon insertion sites against the K-10 complete genome sequence. In mutants 22F4, 30H9 and STM68, the transposon inserted within genes of interest (Figure 5A). In mutant 22F4, the transposon insertion site was mapped internal to MAP0460 (*lsr2*) and located 110 bp from the ATG start codon. For mutant 30H9, transposon insertion sites were mapped as internal to MAP1566. Serendipitously, another screen from a signature-tagged mutagenesis experiment (ca. 300 mutants) identified a Tn*5370* mutant (STM68) with an insertion in the same gene as in 30H9, but the insertion in STM68 was mapped toward the 3′-end coding sequence of MAP1566. STM68 is a stable mutant because Tn*5370* lacks the transposase within the transposed region avoiding the potential for further rearrangements in the mutant strain. In 30H9, Tn*5367* is located 272 bp from the ATG start codon, while mutant STM68 carries a Tn*5370* insertion 51 bp from the TAA termination codon. Thus, for STM68, the insertion may also exert a polar effect on MAP1567 located downstream within the operon. Likewise, though the insertion in 30H9 is well upstream within the MAP1566 coding sequence, a polar effect on MAP1567 cannot be ruled out.

In mutants 2E11, 3H4, and 4H2, the transposon Tn*5367* inserted in the intergenic regions (Figure 5B). For mutant 2E11, Tn*5367* inserted 130 bp upstream from FadE5 and 30 bp upstream from MAP3695. In 3H4, the insertion site was 18 bp upstream from MAP2296c and 182 bp downstream from MAP2297c. In 4H2, the transposon insertion was 39 bp upstream from MAP1150c and 320 bp downstream from MAP1151c. In all these cases, it is hypothesized that the transposon affects the expression of either or both genes adjacent to the insertion sites, or other genes in close proximity. Sequencing of the original isolate of 40A9 putatively identified a transposon insertion site in the intergenic region between MAP0282c and MAP0283c. However, as this insertion point could not be further verified and 7 consecutive nucleotides of primer STM5370-1 matched within MAP0282c sequence, the possibility of spurious priming could not be ruled out. Comparison of the full genomic sequence of 40A9 with K-10 may be necessary to identify any sequence change in this mutant strain. However, this analysis is beyond the scope of this work.

#### Polymerase chain reaction for mutant verification

**Figure 6:**
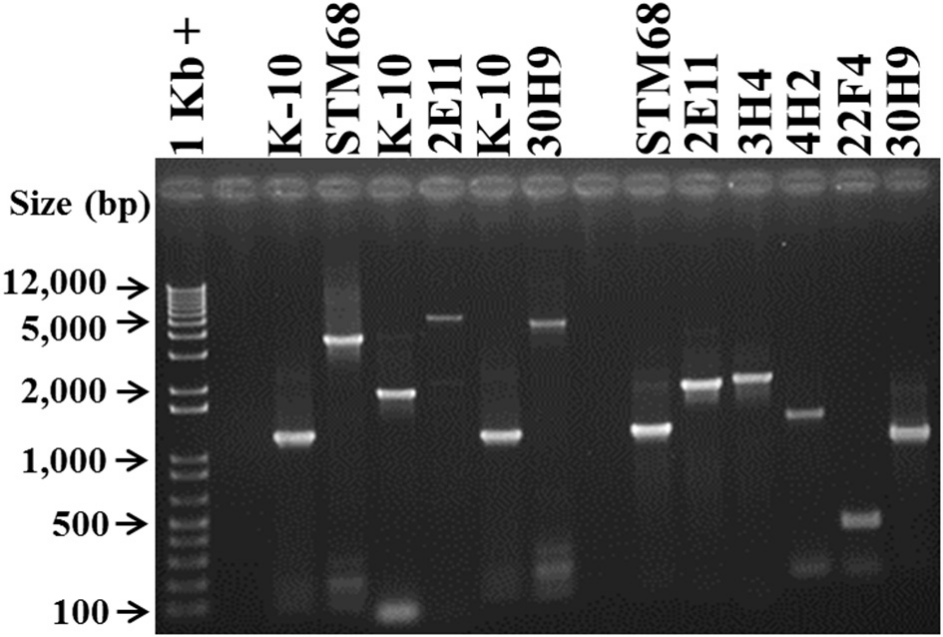
**Analysis of MAP Tn*5367* (3,381 bp) and Tn*5370* (2295 bp) insertion mutants by PCR**. Lane 1, Invitrogen TrackIt™ 1 Kb Plus DNA Ladder; Lanes 2 and 9, empty. Amplicons for the DNA template-primer combinations and corresponding sizes are: Lanes 3 and 7, K-10 with MAP1566-F and MAP1566-R, 1179 bp; Lane 4, STM68 with MAP1566-F and MAP1566-R, 3474 bp; Lane 5, K-10 with 2E11-IP-F and 2E11-IP-R, 1845 bp; Lane 6, 2E11 with 2E11-IP-F and 2E11-IP-R, 5226 bp; Lane 8, 30H9 MAP1566-F and MAP1566-R, 4560 bp; Lane 10, STM68 with MAP1566-F and AMT32, 1160 bp; Lane 11, 2E11 with 2E11-IP-F and AMT32, 1890 bp; Lane 12, 3H4 with 3H4-R and AMT32, 1974 bp; Lane 13, 4H2 with 4H2-F and AMT32, 1431 bp; Lane 14, 22F4 with 22F4-F and AMT32, 561 bp; Lane 15, 30H9 with MAP1566-R and AMT32, 941 bp. Relevant band sizes (bp) for the DNA ladder are displayed to the left.

To demonstrate the presence of specific transposon insertion mutants in mixed infections, such as protection challenge experiments, it was necessary to develop a specific PCR test for each mutant strain. The test is also confirmatory of the sequencing studies to rule out any spurious transposon insertion site identifications. To accomplish this goal, based on the sequencing results, amplicons were synthesized with primers specific for the sequences adjacent to the transposon insertion sites (Table 1), and analyzed on agarose gels (Figure 6). Amplification of wild type K-10 DNA with primers MAP1566-F (within MAP1566 but upstream from the insertion point) and MAP1566-R (within the intergenic region between MAP1566 and MAP1567) resulted in a DNA fragment of 1179 bp (Lanes 3 and 7); these primers yielded fragments of 3474 bp (Lane 4) and 4560 bp (Lane 8), respectively for mutants STM68 and 30H9. These sizes are as expected for the insertion of Tn*5370* (2295 bp) or Tn*5367* (3381 bp), respectively. Likewise, amplification of K-10 (Lane 5) and 2E11 (Lane 6) with primers 2E11-IP-F (within MAP3695) and 2E11-IP-R (within FadE5) resulted in a DNA fragment of 1845 bp and 5226 bp, respectively; thus confirming the predicted insertion of Tn*5367* in the FadE5 and MAP3695 intergenic region.

PCR of mutants 3H4, 4H2, and 22F4 with specific primers (3H4-F, 3H4-R, 4H2-F, 4H2-R, 22F4-F, and 22F4-R, respectively) from regions adjacent to the insertion gave the same amplification product to that of K-10 (unpublished results). However, we were able to confirm the insertion point using a specific primer for the adjacent sequences and primer AMT32 specific for both Tn*5367* and Tn*5370* (McAdam et al., 2002; Shin et al., 2006). In this case, K-10 gave no amplification products (unpublished results). All of the mutant strains gave amplification products of the expected sizes: STM68 (1160 bp, Lane 10), 2E11 (1890 bp, Lane 11), 3H4 (1974 bp, Lane 12), 4H2 (1431 bp, Lane 13), 22F4 (561 bp, Lane 14), and 30H9 (941 bp, Lane 15). We obtained the same amplification product for K-10 and 40A9 using primers 40A9-IP1-F and 40A9-IP1-R adjacent to MAP0283c and MAP0282c (unpublished results). Thus, results confirmed the location of the transposon insertion sites obtained by DNA sequencing. Furthermore, the unique sizes of the PCR amplicons were successfully used to identify the presence of each mutant strain in animal experiments (Bannantine et al., 2014; Hines et al., 2014).

#### Determination of growth curves in broth cultures

**Figure 7:**
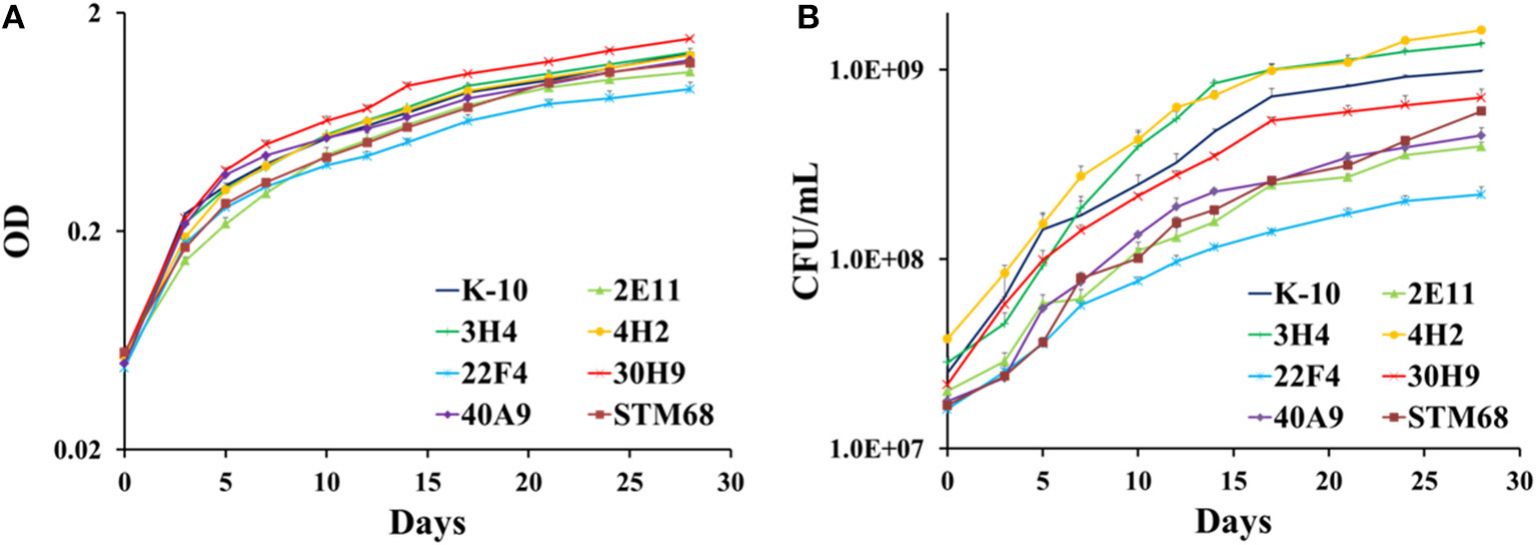
**(A,B) Growth curves of MAP wild type K-10 and mutant strains**. Strains were inoculated into MOADC media at an initial optical density (OD_600_) of approximately 0.05. Cultures were incubated at 37°C standing for 28 days. OD readings **(A)** and CFU after 5 weeks **(B)** were taken. Results represent means (*n* = 3) ± standard error of the mean. See text for relevant *P*-values.

As these MAP mutants were developed as first generation candidate vaccine strains, it was important to determine if their corresponding *in vitro* growth rates in broth cultures were similar to the wild type K-10 strain. Determination and statistical analysis of growth curves indicated that both wild type and mutant strains grew approximately at the same rate in MOADC, as assessed by OD (Figure 7A) or CFU (Figure 7B) determinations. In particular for OD values, growth rates of the mutant strains were not significantly different compared to K-10 (*P* > 0.05), except for 30H9 that grew slightly faster (*P* = 0.047). Likewise the more detailed CFU calculations showed a similar effect, but in this case 3H4 grew faster (*P* = 0.021), 22F4 had a slower rate (*P* = 0.019) and growth of 30H9 did not differ significantly (*P* > 0.05) from K-10. These results suggest that the mutations in these strains are related to properties that relate to virulence such as survival and replication in BoMac cells rather than standard physiological traits. This characteristic is desirable for candidate live-attenuated vaccine strains as the isolates can be readily propagated in broth cultures to prepare inoculums for vaccine delivery.

#### Interaction of wild type and mutant strains with primary bovine macrophages

**Figure 8:**
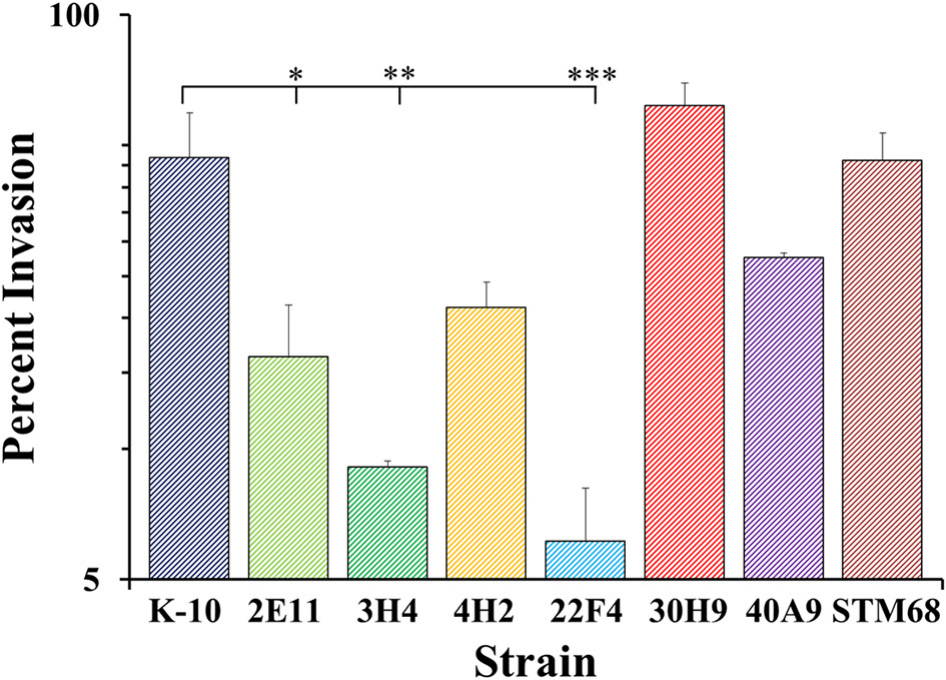
**Invasion of MAP wild type K-10 and mutant strains in bovine macrophages**. Procedures followed are described in Lamont and Sreevatsan (2010). MAP uptake at the end of the invasion incubation period (percent invasion, 2 h after infection). Results represent means (*n* = 3) ± standard error of the mean. Statistical significance for pairwise determinations displayed by a line with tick marks is denoted with asterisks: ^*^P-value < 0.05, ^**^P-value < 0.01 and ^***^P-value < 0.001.

**Figure 9:**
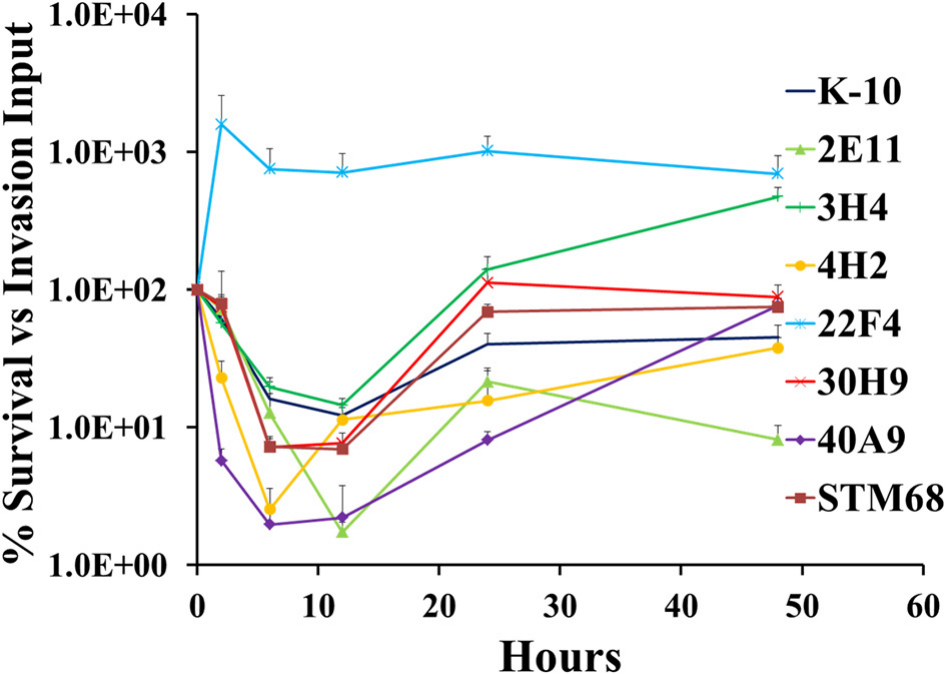
**Survival and replication of MAP wild type K-10 and mutant strains in bovine macrophages**. Procedures followed are described in Lamont and Sreevatsan (2010). Kinetics of MAP intracellular survival and replication (after invasion) are based on the following formula: % Survival = (CFU/ml_time, t_/CFU/ml_time, t = 0_) × 100%; time, *t* is any of the time points while time, *t* = 0 is the time point immediately after completion of invasion incubation. Results represent means (*n* = 3) ± standard error of the mean. See text for relevant *P*-values.

**Figure 10:**
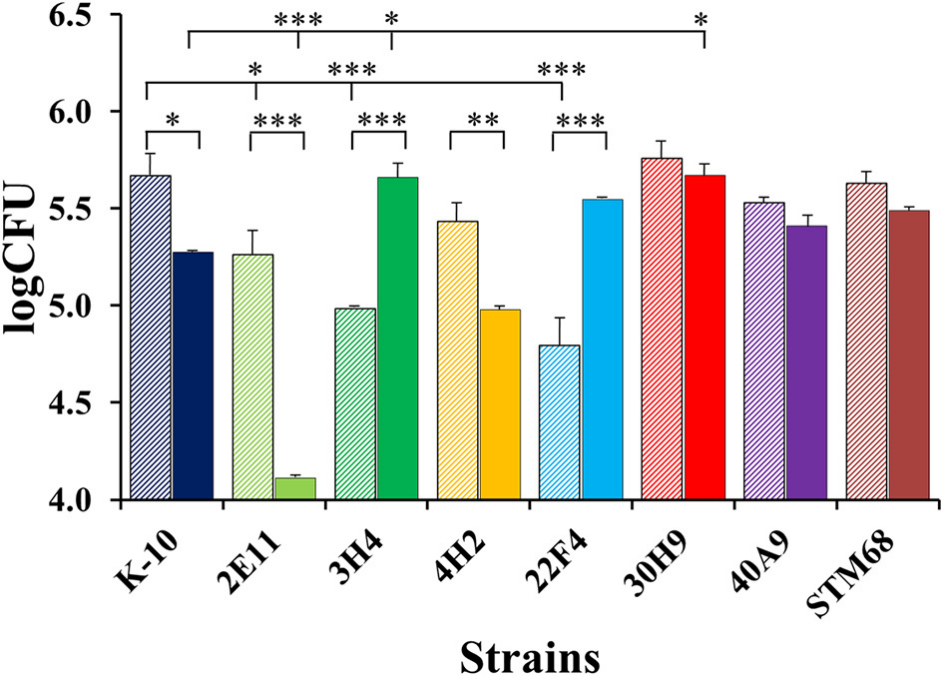
**MAP burdens of wild type K-10 and mutant strains in bovine macrophages**. Procedures followed are described in Lamont and Sreevatsan (2010). Total CFU burdens at Day 0 (2 h after infection, striped bars) and Day 2 (solid bars) are displayed. Results represent means (*n* = 3) ± standard error of the mean. Statistical significance for pairwise determinations displayed by a line with tick marks is denoted with asterisks: ^*^P-value < 0.05, ^**^P-value < 0.01 and ^***^P-value < 0.001.

As macrophages play a key role in MAP infections, to confirm the results obtained with BoMac cells and characterize additional mutants, we determined the interaction of wild type and mutant strains with primary macrophages. MDMs were infected with each strain at a MOI of 10:1, incubated to allow invasion, washed and lysed immediately or at various time points post-infection to determine colony counts (Figures 8–**10**). The results indicated that strains 2E11 (*P* < 0.05), 3H4 (*P* < 0.01) and 22F4 (*P* < 0.001) were significantly less invasive than K-10 (Figure 8). No major differences were observed for all four cases in comparison to K-10 *P* > 0.1. These results sharply contrast with strain invasiveness for BoMac cells (see Section Discussion).

The full short kinetics of the interaction of MAP strains with MDMs is shown in Figure 9. This analysis demonstrates that for most strains there is a pronounced killing phase immediately after invasion incubation up to 6–12 h, followed by a recovery phase and eventual stabilization of the CFU burdens. This aspect of the interaction is usually missed in standard assays that only study 2 time points (immediately after invasion incubation and 2/4 days). In this context, the killing and intracellular replication rates may also influence the final outcome of the interaction of MAP and MDMs. Statistical analysis of each process (e.g., killing and replication-stabilization phases) depicted in these curves was performed. Results indicated that strains 4H2 (*P* < 0.05) and 40A9 (*P* < 0.05) were killed much more rapidly than K-10, while 22F4 (*P* < 0.001) showed an unexpected increase in CFUs during the first 6 h. The intracellular replication rates (evaluated between 12 and 48 h) were similar for the wild type and all mutant strains, except for 3H4 (*P* < 0.01) and 40A9 (*P* < 0.001) that grew faster than K-10.

Figure 10 displays CFU burdens at Day 0 after invasion incubation (striped bars) and Day 2 (solid bars) for mutant and wild type strains. As the MAP inoculums for MDMs were approximately the same for all strains, the CFU burdens at Day 0 are proportional to strain invasiveness (Figure 8). Thus, direct comparisons of Day 0 CFU burdens yielded similar *P* values for pair-wise comparisons as those observed for differences in invasion, except that the decrease in invasiveness for 3H4 (*P* < 0.001) vs. K-10 had a greater significance level. Nonetheless, the analysis based on percent invasion presented in Figure 8 is more accurate, as any differences in the inoculums are taken into account. Regarding strain intracellular survival, as indicated by the CFU burdens for each strain at Day 0 and Day 2, no significant differences were observed for strains 30H9, 40A9, and STM68 (*P* > 0.1). For K-10, the decrease observed in survival is moderate, but falls within statistical significance (*P* < 0.05). In contrast, marked decreases are observed for 2E11 (*P* < 0.001) and 4H2 (*P* < 0.01). Interestingly, 3H4 (*P* < 0.001) and 22F4 (*P* < 0.001) were able to survive and replicate significantly better than K-10 intracellularly. Comparisons of the overall macrophage CFU burdens at Day 2 among all strains allow us to determine the combined effects of invasion and intracellular survival/replication. The results indicate that strain 2E11 is definitively attenuated in comparison to K-10 (*P* < 0.001); while 4H2 is slightly attenuated, but this difference is not statistically significant. Strains 22F4, 40A9, and STM68 behaved similarly to K-10 (*P* > 0.1), while strains 3H4 (*P* < 0.05) and 30H9 (*P* < 0.05) seem to reach higher CFU burdens than K-10.

In summary for the interaction of MAP strains with MDMs, mutants 2E11, 3H4, and 22F4 were less invasive than K-10. In relation to survival, 2E11 and 4H2 displayed marked reductions. For the overall Day 2 CFU burdens, only 2E11 was attenuated. Overall, strains 22F4, 40A9, and STM68 behaved similarly to K-10, while 3H4 and 30H9 reached a higher number of intracellular bacilli. For the killing phase, 4H2 and 40A9 were killed faster than K-10 and all mutant strains were able to grow intracellularly at approximately the same rate, except for 3H4 and 40A9 that replicated faster.

#### Complementation analysis

We also attempted to develop a complementation system for the Tn*5367* transposon mutants using a derivative of the multi-copy plasmid pMV206_Hyg (George et al., 1995). To this end, vector pBUN277 was constructed carrying a *hyg* marker and the green fluorescent protein gene downstream from the mycobacterial heat shock promoter *hsp60*. Assessment of wild type K-10, 4H2 and the empty vector control 4H2 (pBUN277) in MDMs yielded anomalous results. The survival of 4H2 was significantly different (*P* < 0.05) from K-10 at 5 days post-infection, while the survival of 4H2 (pBUN277) was not significantly different (*P* > 0.05) from K-10 (unpublished results). In addition, the survival of 4H2 recombinant strains carrying either the wild type gene MAP1152 or MAP1156 yielded similar results to the empty vector control (*P* > 0.05). This data indicates that the complementation system developed was not appropriate and further attempts were no longer pursued for additional mutants.

## Discussion

The objective of our study was to generate a comprehensive MAP mutant bank and isolate strains with interesting phenotypic properties and identify genomic regions associated with virulence that could be targeted for further development of live-attenuated vaccine strains. Mutants identified for at least one phenotypic property potentially related to virulence are displayed in Table S1. This set of 123 strains represents ca. 1% of the mutant bank. The uniqueness of our approach was to perform the screen independently of homology searches for genes already identified in related mycobacterial species, so as to maximize hits in genes specific for MAP.

Statistical considerations, coupled with the demonstration that randomly picked mutants were independent, suggest that the library was comprehensive: e.g., mutants displayed unique hybridization bands to the IS*1096* probe (see Section Southern hybridization) and diverse phenotypes (Table S1). It is especially noteworthy that five of the mutants selected for the vaccine trail (3H4, 4H2, 22F4, 30H9, and 40A9) and three additional mutants identified as attenuated in BoMac cells or preliminary MDM assays (12G8, 23B5, and 37D1) were all associated with altered DCS susceptibility. As for the rapid screens for cell association, and biofilm and clump formation, five (1B11, 3D9, 4E1, 4E7, and 4F3) out of ca. 300 screened mutants (approximately 1.5%) of the pool, identified strains with properties that may relate to virulence, but did not overlap with the DCS altered susceptibility set. Thus, implementation of additional screens, or expansion of the screens reported herein may still reveal a significant number of potentially attenuated mutants. However, the inherent bias of IS*1096*-derived transposons limits somewhat the comprehensiveness of the mutant bank (McAdam et al., 2002; Shin et al., 2006). Fortunately, *Himar*-derived transposons with more random recognition sequences have been developed for mycobacteria (Sassetti and Rubin, 2003) and are already in use for MAP (Scandurra et al., 2009; Wang et al., 2014). These vectors may provide a more representative mutant repertoire, but at the cost of a significant increase (ca. 10-fold) in the number of mutants that should be screened for a comprehensive analyses. In addition, best recommended screening approaches for *Himar* libraries are focused on gene identification analyses under specified selection pressures (Sassetti and Rubin, 2003). Individual deletion mutants and complementation analysis must then be performed to further confirm the role of these genes in virulence. Thus, the library described herein is useful as source of individual mutants for identification of genes involved in MAP pathogenesis, but implementation of additional screens to formally determine its comprehensiveness may not be warranted. Nonetheless, as described, we performed three comprehensive screens (colony morphology, DCS hypersusceptibility and siderophore production), and three additional limited screens for reduced biofilm and clump formation, and decreased cell association with BoMac cells. Assuming that most of these insertions are in different genes, this collection may represent insertions in ca. 120 genes. In this context, previous studies with MTB demonstrated that 194 genes are required for infection in mice (Sassetti and Rubin, 2003).

Seven Tn*5367* mutants from the set depicted in Table S1 as well as STM68, a mutant from the Tn*5370* signature-tagged mutagenesis pool, were further characterized and their properties are summarized in Table 2. In our comprehensive screening of the mutant bank using a chromogenic assay for siderophore detection, only mutant 1F3 did not display a halo in the CAS plate assay. This mutant grew very slowly in broth cultures but was able to colonize goats (Livneh et al., 2005). Unfortunately, as indicated above, stocks in two laboratories failed to grow upon further attempts at replication and the transposon insertion site could not be determined. The CAS assay phenotype has been associated with impairment of siderophore synthesis or secretion in Msmeg (Fiss et al., 1994). Thus, the properties of 1F3 suggests that MAP has at least an additional siderophore-like molecule that may be involved in iron uptake and play important physiologic roles. Though MAP has a truncated *mbtA* gene that most likely underlies its inability to synthesize mycobactin (Li et al., 2005), mycobactin-dependence is observed mainly in MAP cultured directly from primary lesions or other clinical specimens. In passaged cultures, mycobactin dependence can be readily overcome in acidic iron-rich media (Lambrecht and Collins, 1992). Thus, we hypothesized that 1F3 may have had a lesion in a gene encoding for a siderophore-like molecule other than mycobactin.

**Table 2:** **Screening and characterization of MAP strains**.

**Strain^a^**	**ORF(s)—Mutated or Affected^b^**	**ORFs Function^c^**	**Original Screen and Further Comments^d^**
1F3	Tn*5367* insertion was not identified	Not applicable	Chromogenic screen for siderophore synthesis or transport; mutant lost upon subsequent passages
2E11 Vaccine 316	Tn*5367* between MAP3695 (Rv0245) and FadE5 (Rv0244c)	MAP3695 (hypothetical protein)	Attenuated in MDMs for invasion and intracellular survival
		FadE5 (terpenoid and polyketide metabolism)	
3H4 Vaccine 320	Tn*5367* between MAP2296c (Rv2478c) and MAP2297c (Rv0102)	MAP2296c and MAP2297c (hypothetical proteins)	DCS hypersusceptible mutant chosen as control for normal colony morphology.
			Attenuated for invasion in MDMs, but has faster intracellular replication
4H2 Vaccine 321	Tn*5367* between MAP1150c (Rv3640c) and MAP1151c (Rv2839c)	MAP1150c (IS*1311* transposase)	Colony-morphology mutant, attenuated for invasion in BoMac cells, and invasion and survival in MDMs. Mutant also displayed DCS hypersusceptibility.
	Upstream from MAP1152 (Rv1808)-MAP1156 (Rv1425) cluster	MAP1151c (translational initiation factor IF2)	
		MAP1152 (PPE protein)	
		MAP1156 (diacyglycerol O-acyltransferase)	
22F4 Vaccine 317	Tn*5367* within MAP0460 (Rv3597c, Lsr2)	Lsr2 (H-NS like iron-regulated DNA binding protein that also provides protection against oxidative stress)	DCS hypersusceptible; attenuated for survival in BoMac cells and invasion in MDMs
30H9 Vaccine 319	Tn*5367* within MAP1566 (Rv1645c) near 5′-end coding sequence	MAP1566	DCS hypersusceptible; attenuated for invasion in BoMac cells
		ORF downstream from ModA (MAP1565) in the mod operon	
40A9 Vaccine 318	Tn*5367* between MAP0282c (Rv3860) and MAP0283c (Rv2082)	MAP0282c and MAP0283c (hypothetical proteins, assigned with low confidence); or spontaneous rRNA mutations	Decreased susceptibility to DCS; transposon lost upon further passage; attenuated in MDMs by increased killing rate
STM68 Vaccine 315	Tn*5370* within MAP1566 (Rv1645c) near 3′-end coding sequence	MAP1566	Identified as MAP1566 insertion by direct sequencing of Tn*5370* signature-tagged mutagenesis pool
		ORF downstream from ModA (MAP1565) in the *mod* operon	

a *Laboratory designation followed by the vaccine trial designation (Bannantine et al., 2014; Hines et al., 2014)*.

b *MAP ORF designation is followed by MTB definite or putative ortholog in parenthesis*.

c *See text for citations on ORFs functions as applicable*.

d *See text for further details*.

Mutant 2E11 was a false positive for reduced biofilm formation, but it was nonetheless selected for further studies, since preliminary assays indicated attenuation in MDMs. Assignment of the target gene for mutant 2E11 was not fully resolved in this study as the Tn*5367* insertion was mapped to the intergenic region (Figure 5) encoding an acyl-coA dehydrogenase (MAP3694c, aka FadE5) and a possible oxidoreductase (MAP3695). Interestingly, FadE5 is involved in lipid degradation (http://www.ncbi.nlm.nih.gov/biosystems/1743) including the metabolism of terpenoids and polyketides, such as geraniol, and these compound types have been associated with biofilm formation in bacteria (Zheng et al., 2013). MAP FadE5 is orthologous to MTB Rv0244c, which has been shown to be required for *in vitro* growth on cholesterol (http://tuberculist.epfl.ch). This mutant was the most attenuated in this study on account of both invasiveness and survival in MDMs.

Mutant 3H4 displayed a transposon insertion in the intergenic region between MAP2296c and MAP2297c. Both these ORFs encode for hypothetical proteins, with the MTB ortholog (Rv2478c) possibly encoding a non-essential DNA binding protein, and MAP2297c ortholog (Rv0102) identified as an integral essential membrane protein and ABC transporter that may underlie the DCS hypersusceptible phenotype (http://tuberculist.epfl.ch). Though, this mutant was originally selected as a normal colony morphotype control, it displayed hypersusceptibility to DCS. Nonetheless, this strain behaved similarly to the wild type K-10 strain in both BoMac cells and MDMs, except that it displayed an attenuated invasion phenotype in the interaction with MDMs that was compensated with a higher intracellular replication rate.

In 4H2, the transposon inserted within the intergenic region between MAP1150c and MAP1151c. MAP1150c is the transposase for IS*1311* (Murcia et al., 2007), an insertion sequence present at eight copies in the MAP genome (Li et al., 2005). Thus, any effect of this insertion on the MAP1150c is not likely to have a major impact on MAP physiology. In addition, the insertion may not have an effect on MAP1151c either as the transposon is located beyond the 3′-end of this coding sequence. However, there is a gene cluster (MAP1152-MAP1156) (Bannantine et al., 2011) that is transcribed from the complementary strand to MAP1150c, whose expression could be effected by the transposon insertion that is located 634 bp upstream from the 5′-end of MAP1152. This gene cluster is very interesting since MAP1152, MAP1153 and MAP 1155 encode PPE proteins of the ancestral sublineage IV (Gey Van Pittius et al., 2006). PPE proteins have been suggested to play a role as host range determinants (Cole et al., 1998) and the immunopathogenesis of both humoral and cellular responses in MTB (Choudhary et al., 2003; Okkels et al., 2003) and MAP (Nagata et al., 2005). Other PPE proteins in MTB (Rv1807, Rv3872, and Rv3873) have been implicated in virulence since inactivation of the corresponding genes results in mutants attenuated in mice (Sassetti and Rubin, 2003). In addition, PPE and PE proteins function in pairwise combinations of interacting partners exposed to the cell surface (Riley et al., 2008) and both sets are markedly underrepresented in MAP (cf. 36 PPE proteins in MAP vs. 68 in MTB) (Li et al., 2005). The other members of the gene cluster, MAP1154 and MAP1156, encode proteins of an uncharacterized protein family. MAP1154 encodes a hypothetical protein, while MAP1156 has putative diacyglycerol O-acyltransferase activity (Bannantine et al., 2011). Mutant 4H2 was clearly attenuated in both BoMac and MDM cells, on account of reduced invasiveness and intracellular survival. Moreover, we have demonstrated that MAP1152 and MAP1156 engender humoral immunity responses that can be used to identify infected animals and thus, the corresponding mutants have potential for DIVA (differentiating infected from vaccinated animals) vaccine formulations (Bannantine et al., 2011).

Mutants 22F4 and 30H9 were identified as DCS hypersusceptible strains. Isolate 22F4 carries an insertion within the gene encoding the Lsr2 protein that has been implicated in various roles: dominant T cell responses in MTB (Stewart et al., 2002) and biofilm formation and colony morphology in Msmeg (Chen et al., 2006). Furthermore, a MAP *lsr2* deletion mutant has been constructed (Park et al., 2008) that showed similar properties and a marginal degree of attenuation in MDMs to 22F4 (Lamont et al., 2014), thus providing strong evidence that the transposon insertion, that is internal to *lsr2*, does disrupt the function of this gene.

Strain 30H9 has an insertion internal to MAP1566 and proximal to its 5′-end coding sequence. Likewise, STM68 carries an insertion in MAP1566 but closer to its 3′-end that could potentially have a greater effect on the downstream gene MAP1567. The cluster MAP1565-MAP1568 seems to comprise an operon encoding a high affinity molybdate transport system, as indicated by bioinformatic analysis (Braibant et al., 2000). Orthologs are present in *M. avium* strain 104 (MAV_2862, 99% identity), MTB (Rv1645c, 35% identity, 50% similarity) as well as other mycobacterial species. However, only the MAP and *M. avium* 104 strain orthologs are located within the Mod operon, making this arrangement unique to these species. We hypothesize that MAP1566 could be a chaperone that works in concert with this transport system, while MAP1565 (ModA) and MAP1567 (ModB) are integral membrane proteins that constitute and stabilize the transport channel. It seems that MAP1569 (ModD) has a function in fibronectin binding unrelated to this molybdenum transport system (Kumar et al., 2003) and thus it may correspond to a different transcriptional unit. Strong evidence for the transposon insertions affecting the functions of Mod operon (e.g., MAP1566 or MAP1567) is provided by the similar properties of both mutants in MDMs, mice (Bannantine et al., 2014) and goats (Hines et al., 2014). These mutants were attenuated in C57BL/6 mice but protective against wild type challenge, displayed low skin reactivity in goats and showed significant attenuation in MDM assays as analyzed elsewhere (Lamont et al., 2014). However, in this current analysis, 30H9 and STM68 were comparable to the wild type strain. Interestingly, another MAP1566 Tn*5367* mutant (WAg906), derived from the MAP wild type strain 989 (Cavaignac et al., 2000), was shown to be attenuated based on the average slope values obtained in long-term MDM assays as well as BALB/c mice at 12 weeks post-infection (Scandurra et al., 2010; Bannantine et al., 2014; Lamont et al., 2014). However, in protection challenge experiments, 30H9 and STM68 showed good protection in C57BL/6 mice against wild type challenge, while WAg906 failed to protect BALB/c mice. Use of different MAP and mouse strains may explain these results. These three mutants were attenuated for survival in goats, but only 30H9 and STM68 were tested for protection against challenge, displaying low efficacy (Scandurra et al., 2010; Hines et al., 2014).

As for 40A9, the loss of the transposon insertion does not allow us to make any definitive conclusions on the molecular basis underlying its attenuation. However, this mutant still retained the Kan-resistant phenotype suggesting the involvement of spontaneous mutations in ribosomal RNA genes (Du et al., 2013). Nonetheless, this mutant was the most protective from this collection in the goat trial (Hines et al., 2014). The most interesting property observed for this strain was a fast intracellular killing in MDMs followed by its ability to recover and grow (Figure 9), but otherwise this strain reached a similar CFU burden as K-10 at Day 2 post-infection (Figure 10).

Regarding the complementation studies, there are several reasons that may explain the anomalous results including: (i) the *hyg* marker may increase the survival of the host strains as it was observed for Msmeg recombinant strains (Chacon et al., 2009); (ii) the inherent instability of the Tn*5367* mutant strains with the potential for further transposition events by a cut and paste mechanism (McAdam et al., 1995); (iii) the possibility of spurious recombination events for strains carrying multiple copies of wild type DNA sequences in the complementing plasmids and the transposon interrupted copies in the genome. Due to these difficulties, conclusions regarding the roles of the genes identified in this study are still presumptive rather than definitive. Nonetheless, the bioinformatic analysis and the correlation with our previous results and those from other laboratories increase the confidence that most of these genes are relevant to MAP survival and pathogenesis. Moreover, MAP slow growth and low transformation efficiency place an onerous time demand on further attempts to revisit complementation studies (Foley-Thomas et al., 1995). These issues may underlie the lack of reported complementation analysis in previous MAP transposon mutant reports (Shin et al., 2006; Scandurra et al., 2009, 2010). To overcome these complications, we are currently implementing more solid strategies to construct and complement deletion mutants using different selection markers.

An important objective of this study was to compare the interaction of different MAP strains with BoMac cells and MDMs in this study and the parallel study in the vaccine trial (Lamont et al., 2014). In our studies, the order of attenuation in BoMac cells and MDM cultures (comparing invasion percentiles and slopes for each infection phase) was significantly different. Strain 30H9 displayed the greatest attenuation in BoMac cells, while it was the least attenuated in the MDM assays based on the analysis reported herein. Mutants 3H4 and 22F4 yielded less discrepancy, while 4H2 behaved similarly. However, when the strains were compared only by the average slope values in MDM assays (Lamont et al., 2014), results were closer to those obtained with BoMac cells, with 30H9 yielding the greatest attenuation among the four strains that were also tested in BoMac cells. Notably, 3H4, 22F4, and 30H9 had major differences in invasiveness for the two cell types: 3H4 and 22F4 were invasive for BoMac cells, but not for MDMs (Figures 3, 8), while the opposite was observed for 30H9. The differences observed are likely due to the inherent nature of the BoMac cell line that has poor phagocytosis and decreased ability to sustain intracellular MAP replication, as shown previously for the wild type strain K-10 (Woo et al., 2006). A similar situation seems to occur in MDM aging cultures (Scandurra et al., 2010; Lamont et al., 2014). Thus, determination of the early kinetics at each phase (e.g., invasion, intracellular killing and replication), as done in this study, seems to provide the best assessment of strain attenuation for studies in ruminants. This analysis may be supplemented with assays to determine apoptosis and measure cytokines such as IL-10 (Scandurra et al., 2010; Kabara and Coussens, 2012; Lamont et al., 2014).

Comparing the results from this study with the recent vaccine trial, though not conclusive, sheds some light on the relation among the various models and vaccine protection for ruminants. Attenuation in BoMac cells and analysis of average slopes in MDM assays showed significant correlation with attenuation and protection in C57BL/6 mice vaccinated by intraperitoneal injection, but not with orally vaccinated goats (Bannantine et al., 2014; Hines et al., 2014; Lamont et al., 2014). In contrast, the analysis in this manuscript of MDM assays correlated well with protection results in goats. Indeed, the four strains tested in goats ranked similarly; except for the inversion between 2E11 (most attenuated in MDMs as reported here, but ranked 2nd in goats) and 40A9 (ranked 2nd in MDMs, but most protective in goats).

As to the MAP mutant properties necessary to elicit protective immunity in ruminants, based on the conditions tested in the vaccine trial, interplay between attenuation and virulence seems necessary. Moreover, a level of persistence (e.g., able to colonize well *in vivo* at early time points but lead to significant reduction in colonization at later times post-infection) has been hypothesized as one desirable property for the design of MTB live attenuated vaccines (Hingley-Wilson et al., 2003). For example, 40A9 would be considered rather wild type like, based on the CFU burdens at either Day 0 or Day 2 (Figure 10). However, this strain displays both faster killing and intracellular replication rates (Figure 9). Significant killing of 40A9 in their first encounter with macrophages may provide sufficient attenuation while preserving protective immunogenicity. The subsequent fast intracellular recovery may lead to a persistent behavior. Though, 40A9 was not tested in BALB/c mice for the persistent phenotype, this behavior was observed for Vaccine 329 that outperformed 40A9 in goats (Shin et al., 2006; Hines et al., 2014). In contrast, 30H9 and STM68 displayed a behavior similar to the wild type K-10 in each of the steps involved in the interaction of the mutant strains with MDMs (Figures 8–10), but performed worse in the goat vaccination trial. In addition, little correlation was observed between the mouse and goat trails, though in this case the more susceptible C57BL/6 mice were used (Bannantine et al., 2014). Thus, the level of protection of a candidate vaccine strain may depend on earlier interactions of the mutant strains with the oral, gastric and intestinal mucosa, and intracellular killing and replication rates in MDMs (Bermudez et al., 2010). In addition, there still remains significant issues regarding tissue culture (BoMac vs. MDM cells), analysis of tissue culture results, animal model (BALB/c and C57BL/6 mice or goats), dose (low vs. high) and routes of inoculation (intraperitoneal, oral and subcutaneous) that need further testing and standardization to make better predictions on protection for JD vaccines in cattle.

## Conclusions

The major accomplishments and findings of this study are: (1) a comprehensive MAP Tn*5367* transposon mutant bank was constructed by indexing a large collection of independent mutants that was statistically representative of the full genome; (2) Tn*5367* mutants were inherently unstable due to the IS*1096* transposase presence within the transposed sequence; (3) phenotypic screens were effective in identifying attenuated mutants or processes of importance for pathogenesis or cell physiology: (a) the presence of additional siderophores other than mycobactin (e.g., 1F3), (b) the association of colony morphotypes with reduced virulence (e.g., 4H2 and 22F4), (c) the association of drug-susceptible (e.g., 22F4 and 30H9) or resistant (e.g., 4H2 and 40A9) phenotypes with reduced virulence and (d) the potential role of cell association, and biofilm and clump formation with virulence (see Section Miscellaneous screening); (4) mutant screening in BoMac cells and MDMs yields significantly different results; (5) strain invasiveness and slope values at each step in the interaction of MAP bacilli with MDMs, especially the slopes during the early killing phase, are the best predictors for protection against wild type challenge in the oral goat vaccination model; (6) the interaction of MAP with MDMs is complex and key aspects of this process involve: invasion, intracellular killing, intracellular replication and maintenance of CFU intracellular burdens; (7) mutant screening with BoMac cells may have significant biases and it should be abandoned as a routine methodology to assess attenuation; and (8) the most important stages of MAP interaction with MDMs in tissue culture occur at relatively short times (up to 48 h post-invasion incubation). Our results suggests that further studies with MAP mutants should focus on early step-wise analysis of their interactions with MDMs and the use of transposon delivery vectors that lack the transposase within the transposed element (e.g., Tn*5370* and the newly developed *Himar1* derivatives).

### Conflict of interest statement

Patent: Methods for the identification of Virulence Determinants. Raúl G. Barletta, N. Beth Harris. U.S. Patent No. 7,740,867, Granted June 22, 2010. The Review Editor, Jeffrey Cirillo, declares that, despite having collaborated with authors Raul Barletta and Denise Kinniel, the review process was handled objectively. The authors declare that the research was conducted in the absence of any commercial or financial relationships that could be construed as a potential conflict of interest.

## Supplementary material

Figure S1 **(A–D) Screening of MAP mutants for susceptibility or resistance to DCS**. All mutants were plated on MOADC-Plus media without **(A,C)** and with **(B,D)** DCS at 20 μg/ml and allowed to grow for 8 weeks. There are two different scale bars displayed since a Fisherbrand 08-757-14 round **(A,C)** or Corning 431110 square **(B,D)** plate was utilized. Mutants with altered DCS susceptibility are circled with black outlines: the rough colony morphotype 4H2 **(A,B)** displays a resistant phenotype while the smooth colony morphotype 22F4 **(C,D)** displays hypersusceptibility.

Figure S2 **Rapid screening for MAP mutants with decreased biofilm formation**. The left image depicts a MAP mutant with reduced biofilm formation while the image on the right is a mutant with normal biofilm formation.

Figure S3 **(A,B) Rapid screening for MAP mutants with reduced cell association**. A rapid *in vitro* assay was developed to screen the library for mutants with decreased cell association (e.g., less adherence and/or invasion) with BoMac cells. Cells were plated on a 16-well chamber slide and infected with MAP wild type and mutant strains. At 24 h post-incubation, slides were fixed and acid-fast bacteria were stained by the Auramine-Rhodamine method that yields green fluorescence for acid-fast bacilli. Representative microscopic images from an infection with the wild type strain K-10 **(A)** and a mutant with decreased cell association **(B)** are shown. Internal scale bar is 100 μm.

Figure S4 **(A,B) Rapid screening for MAP mutants with reduced clump formation**. To test for mutants with reduced clump formation, a property that could be related to virulence, broth cultures were analyzed by microscopy. Microscopic images of the wild type strain K-10 **(A)** and a mutant with reduced clump formation **(B)** are shown. Internal scale bar is 10 μm.

## Abbreviations

CAS: Chrome azurol S
CFU: Colony forming units
DCS: D-cycloserine
Hyg: Hygromycin
JD: Johne's Disease
Kan: Kanamycin
MAP: *Mycobacterium avium* subsp. *paratuberculosis*
MDM: Monocyte-derived macrophage
MOADC: Middlebrook 7H9 broth enriched with oleic acid albumin dextrose complex
Msmeg: *Mycobacterium smegmatis*
MTB: *Mycobacterium tuberculosis*
OD: Optical density
ORF: Open reading frame
PBS: Phosphate buffered saline
SEM: Standard error of the mean.
