# Supplementary figures and images for: Generation and screening of a comprehensive Mycobacterium avium subsp. paratuberculosis transposon mutant bank
Source: Front Cell Infect Microbiol. 2014 Oct 15;4:144. doi: 10.3389/fcimb.2014.00144 (PMC4197770; doi:10.3389/fcimb.2014.00144)

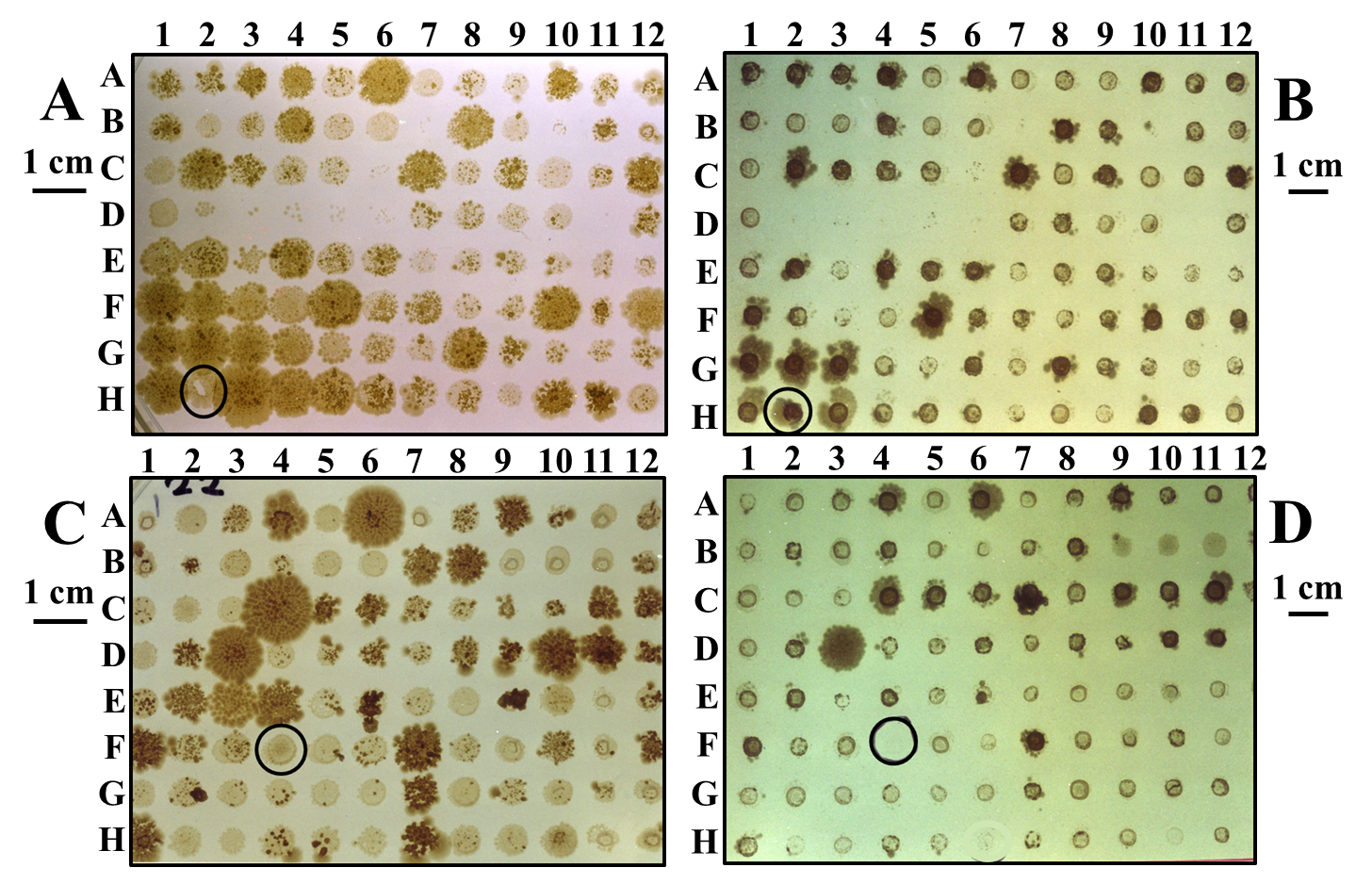

Supplement: Figure S1 — (A–D) Screening of MAP mutants for susceptibility or resistance to DCS. All mutants were plated on MOADC-Plus media without (A,C) and with (B,D) DCS at 20 μg/ml and allowed to grow for 8 weeks. There are two different scale bars displayed since a Fisherbrand 08-757-14 round (A,C) or Corning 431110 square (B,D) plate was utilized. Mutants with altered DCS susceptibility are circled with black outlines: the rough colony morphotype 4H2 (A,B) displays a resistant phenotype while the smooth colony morphotype 22F4 (C,D) displays hypersusceptibility. [file Image1.TIF]

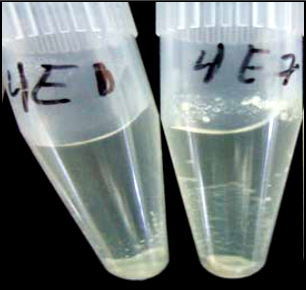

Supplement: Figure S2 — Rapid screening for MAP mutants with decreased biofilm formation. The left image depicts a MAP mutant with reduced biofilm formation while the image on the right is a mutant with normal biofilm formation. [file Image2.TIF]

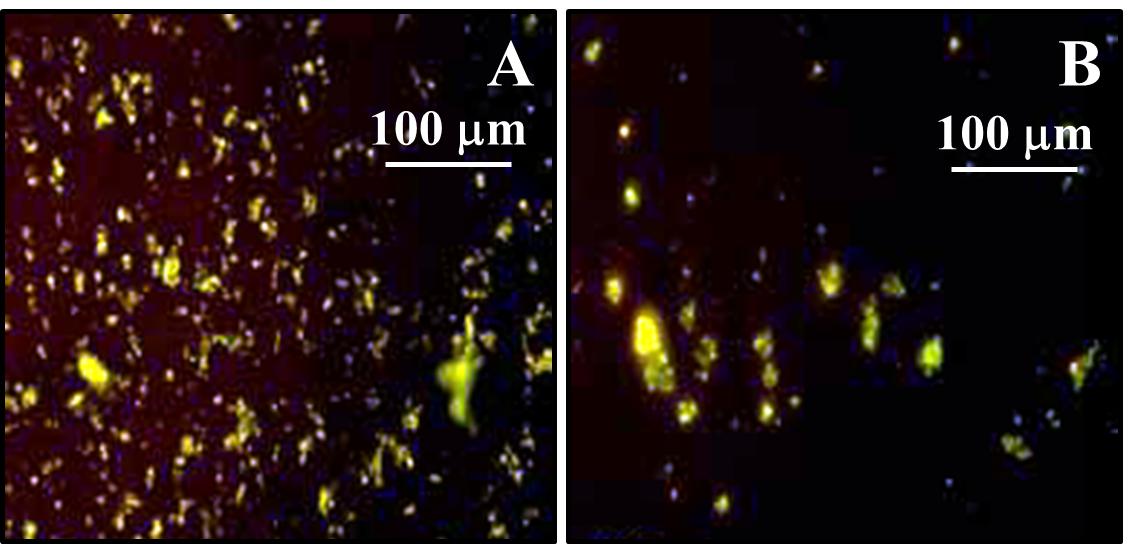

Supplement: Figure S3 — (A,B) Rapid screening for MAP mutants with reduced cell association. A rapid in vitro assay was developed to screen the library for mutants with decreased cell association (e.g., less adherence and/or invasion) with BoMac cells. Cells were plated on a 16-well chamber slide and infected with MAP wild type and mutant strains. At 24 h post-incubation, slides were fixed and acid-fast bacteria were stained by the Auramine-Rhodamine method that yields green fluorescence for acid-fast bacilli. Representative microscopic images from an infection with the wild type strain K-10 (A) and a mutant with decreased cell association (B) are shown. Internal scale bar is 100 μm. [file Image3.TIF]

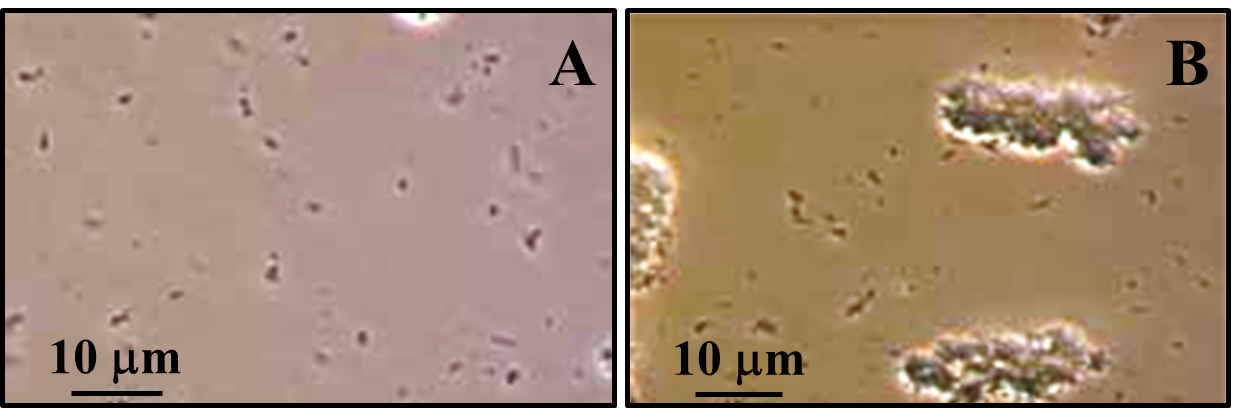

Supplement: Figure S4 — (A,B) Rapid screening for MAP mutants with reduced clump formation. To test for mutants with reduced clump formation, a property that could be related to virulence, broth cultures were analyzed by microscopy. Microscopic images of the wild type strain K-10 (A) and a mutant with reduced clump formation (B) are shown. Internal scale bar is 10 μm. [file Image4.TIF]
