# Supplementary material for: Generation and screening of a comprehensive Mycobacterium avium subsp. paratuberculosis transposon mutant bank
Source: Front Cell Infect Microbiol. 2014 Oct 15;4:144. doi: 10.3389/fcimb.2014.00144 (PMC4197770; doi:10.3389/fcimb.2014.00144)
Supplement: Supplementary file 5 [file Table1.PDF]

Table S1 | MAP Tn*5367* mutants with properties related to attenuation.

Mutant Strains[1]	DCS Susceptibility	Colony Morphology	Additional Comments
**1F3**	No Growth	Altered	Positive for IS*1096* Southern Hybridization; Impaired in Siderophore Production or Transport
1G8	Poor Growth	Normal	Positive for IS*1096* Southern Hybridization
2D10	No Growth	Normal	Positive for IS*1096* Southern Hybridization
**2E11**	Normal Growth	Normal	Positive for IS*1096* Southern Hybridization; Biofilm False Positive; Attenuated in MDMs
3B11	Normal Growth	Normal	Decreased Cell Association
3D4	Poor Growth	Normal	Decreased Cell Association
3D9	Normal Growth	Normal	Decreased Clump Formation
3E5	No Growth	Normal	Positive for IS*1096* Southern Hybridization
**3H4**	No Growth	Normal	Positive for IS*1096* Southern Hybridization; Reduced Invasion in MDMs
4E1	Normal Growth	Normal	Decreased Biofilm Formation
4E7	Normal Growth	Normal	Decreased Biofilm and Clump Formation
4F3	Normal Growth	Normal	Increased Biofilm Formation
**4H2**	Fast Growth	Altered	Positive for IS*1096* Southern Hybridization; Attenuated in BoMac Cells and MDMs
5E2	Poor Growth	Normal	Positive for IS*1096* Southern Hybridization
5E5	Poor Growth	Normal	Negative for IS*1096* Southern Hybridization
5E6	Poor Growth	Normal	Positive for IS*1096* Southern Hybridization
6G9	No Growth	Normal	No Further Testing
6G12	Poor Growth	Normal	No Further Testing
7D11	Poor Growth	Normal	No Further Testing
7E7	Poor Growth	Normal	Positive for IS*1096* Southern Hybridization
7F3	No Growth	Normal	No Further Testing
10D4	No Growth	Normal	No Further Testing
10H5	No Growth	Normal	No Further Testing
12B4	Poor Growth	Normal	Positive for IS*1096* Southern Hybridization
12G8	No Growth	Normal	Positive for IS*1096* Southern Hybridization; Attenuated in BoMac Cells
13E1	No Growth	Normal	Positive for IS*1096* Southern Hybridization
15A6	Fast Growth	Normal	No Further Testing
16A10	Poor Growth	Normal	No Further Testing
16B11	No Growth	Altered	No Further Testing
16C6	Fast Growth	Normal	Positive for IS*1096* Southern Hybridization
17H3	Poor Growth	Normal	No Further Testing
19E1	Poor Growth	Normal	Positive for IS*1096* Southern Hybridization
20F5	Fast Growth	Normal	Positive for IS*1096* Southern Hybridization

21G4	Poor Growth	Normal	Positive for IS*1096* Southern Hybridization
21G8	Poor Growth	Normal	No Further Testing
**22F4**	No Growth	Altered	Positive for IS*1096* Southern Hybridization; Attenuated in BoMac and MDMs
22H6	Poor Growth	Normal	Positive for IS*1096* Southern Hybridization
22H10	Poor Growth	Normal	No Further Testing
23B5	Poor Growth	Normal	Positive for IS*1096* Southern Hybridization; Attenuated in MDMs
23B6	No Growth	Normal	No Further Testing
24A10	Poor Growth	Normal	Positive for IS*1096* Southern Hybridization
25B8	Poor Growth	Normal	No Further Testing
24C5	Poor Growth	Normal	No Further Testing
30G8	No Growth	Normal	Positive for IS*1096* Southern Hybridization
**30H9**	No Growth	Normal	Positive for IS*1096* Southern Hybridization; Attenuated in BoMac Cells
31D3	No Growth	Normal	Positive for IS*1096* Southern Hybridization
32F6	No Growth	Normal	No Further Testing
33F3	Poor Growth	Normal	No Further Testing
34C5	Normal Growth	Normal	Positive for IS*1096* Southern Hybridization
35E7	No Growth	Normal	No Further Testing
36E5	No Growth	Normal	Positive for IS*1096* Southern Hybridization
37C12	No Growth	Normal	Positive for IS*1096* Southern Hybridization
37D1	No Growth	Normal	Positive for IS*1096* Southern Hybridization; Attenuated in BoMac Cells
38F6	No Growth	Normal	No Further Testing
39H8	No Growth	Normal	Positive for IS*1096* Southern Hybridization
**40A9**	Fast Growth	Normal	Positive, then Negative for IS*1096* Southern Hybridization; Increased Killing Rate in MDMs
41E7	No Growth	Normal	No Further Testing
42F3	No Growth	Normal	Positive for IS*1096* Southern Hybridization
44A2	Fast Growth	Normal	No Further Testing
44E2	Fast Growth	Normal	No Further Testing
45F10	No Growth	Normal	No Further Testing
47C4	No Growth	Normal	No Further Testing
47D1	No Growth	Normal	Positive for IS*1096* Southern Hybridization
57D7	Fast Growth	Normal	No Further Testing
61C8	No Growth	Normal	No Further Testing
65E9	Fast Growth	Altered	No Further Testing
69D12	Fast Growth	Altered	No Further Testing
71G11	No Growth	Normal	No Further Testing
73D7	Normal Growth	Altered	No Further Testing
79G12	Fast Growth	Normal	No Further Testing

84D12	Normal Growth	Altered	No Further Testing
97E2	Fast Growth	Normal	No Further Testing
98E2	No Growth	Normal	No Further Testing
98G12	Poor Growth	Normal	No Further Testing
99B6	No Growth	Normal	No Further Testing
99C6	No Growth	Normal	No Further Testing
99E4	No Growth	Normal	No Further Testing
99E5	No Growth	Normal	No Further Testing
100C3	No Growth	Normal	No Further Testing
100D10	No Growth	Normal	No Further Testing
100F10	No Growth	Normal	No Further Testing
100H12	Fast Growth	Normal	No Further Testing
101A1	Poor Growth	Normal	No Further Testing
101B6	Poor Growth	Normal	No Further Testing
101H4	Poor Growth	Normal	No Further Testing
102A2	Poor Growth	Normal	No Further Testing
102A6	Poor Growth	Normal	No Further Testing
102F4	Fast Growth	Normal	No Further Testing
102G1	Poor Growth	Normal	No Further Testing
103E10	Fast Growth	Normal	No Further Testing
105B2	No Growth	Normal	No Further Testing
107B7	No Growth	Normal	No Further Testing
114B6	No Growth	Normal	No Further Testing
114D6	Poor Growth	Normal	No Further Testing
114E2	Poor Growth	Normal	No Further Testing
119D2	Poor Growth	Normal	No Further Testing
119D6	Poor Growth	Normal	No Further Testing
120E8	No Growth	Normal	No Further Testing
122A11	No Growth	Normal	No Further Testing
122D2	No Growth	Normal	No Further Testing
122D6	No Growth	Normal	No Further Testing
123D9	No Growth	Normal	No Further Testing
123G7	No Growth	Normal	No Further Testing
125A6	Normal Growth	Normal	Positive for IS*1096* Southern Hybridization
125D2	No Growth	Normal	No Further Testing
126F5	No Growth	Normal	No Further Testing
126F8	No Growth	Normal	No Further Testing
126F9	No Growth	Normal	No Further Testing
127A3	No Growth	Normal	No Further Testing
130E5	No Growth	Normal	No Further Testing
130E12	No Growth	Normal	No Further Testing

130G6	No Growth	Normal	No Further Testing
132B11	No Growth	Normal	No Further Testing
132G1	No Growth	Normal	No Further Testing
133E8	No Growth	Normal	No Further Testing
133G3	No Growth	Normal	No Further Testing
133G8	No Growth	Normal	No Further Testing
133G10	No Growth	Normal	No Further Testing
134C2	Poor Growth	Normal	No Further Testing
134C3	Poor Growth	Normal	No Further Testing
134C4	Poor Growth	Normal	No Further Testing
134G9	No Growth	Normal	No Further Testing
136E6	No Growth	Normal	No Further Testing

[1]MAP mutants that are bolded are further described in Table 2.
